# CLIP-based prediction of mammalian microRNA binding sites

**DOI:** 10.1093/nar/gkt435

**Published:** 2013-05-22

**Authors:** Chaochun Liu, Bibekanand Mallick, Dang Long, William A. Rennie, Adam Wolenc, C. Steven Carmack, Ye Ding

**Affiliations:** Wadsworth Center, New York State Department of Health, Center for Medical Science, 150 New Scotland Avenue, Albany, NY 12208, USA

## Abstract

Prediction and validation of microRNA (miRNA) targets are essential for understanding functions of miRNAs in gene regulation. Crosslinking immunoprecipitation (CLIP) allows direct identification of a huge number of Argonaute-bound target sequences that contain miRNA binding sites. By analysing data from CLIP studies, we identified a comprehensive list of sequence, thermodynamic and target structure features that are essential for target binding by miRNAs in the 3′ untranslated region (3′ UTR), coding sequence (CDS) region and 5′ untranslated region (5′ UTR) of target messenger RNA (mRNA). The total energy of miRNA:target hybridization, a measure of target structural accessibility, is the only essential feature common for both seed and seedless sites in all three target regions. Furthermore, evolutionary conservation is an important discriminating feature for both seed and seedless sites. These features enabled us to develop novel statistical models for the predictions of both seed sites and broad classes of seedless sites. Through both intra-dataset validation and inter-dataset validation, our approach showed major improvements over established algorithms for predicting seed sites and a class of seedless sites. Furthermore, we observed good performance from cross-species validation, suggesting that our prediction framework can be valuable for broad application to other mammalian species and beyond. Transcriptome-wide binding site predictions enabled by our approach will greatly complement the available CLIP data, which only cover small fractions of transcriptomes and known miRNAs due to non-detectable levels of expression. Software and database tools based on the prediction models have been developed and are available through Sfold web server at http://sfold.wadsworth.org.

## INTRODUCTION

MicroRNAs (miRNAs) are an abundant class of small endogenous non-coding RNAs (ncRNAs) of ∼22 nucleotides (nt) in length that have been found in plants, animals and viruses. miRNAs are post-transcriptional regulators involved in the regulation of diverse developmental processes, molecular and cellular pathways and human diseases (1). A mature miRNA can guide RNA-induced silencing complex (RISC) for target recognition by sequence complementarity between the miRNA and sequences typically in the 3′ untranslated regions (3′ UTRs) of the cognitive messenger RNAs (mRNAs). Successful target binding usually results in translational repression and/or mRNA degradation (2). Each human miRNA is believed to be able to regulate several hundred different mRNAs (3). On the other hand, an individual mRNA may be simultaneously targeted by multiple miRNAs. These highlight the versatility and extensiveness of gene regulation by miRNAs. For animal miRNAs, the complementarity is typically imperfect with mismatches. This presents a major challenge for target identification, as straightforward sequence-alignment-based genome-scale target search generates too many hits to be useful for efficient experimental validation.

Experimental validation of computational predictions has been the most popular and simplest approach for target identification. Most of the existing algorithms are based on the seed rule, i.e., the target site within 3′ UTR forms Watson–Crick (WC) pairs with bases at positions 2 through 7 or 8 of the 5′ end of the miRNA (3). However, numerous exceptions to the seed rule have been well documented (4–7). Other sequence features have been proposed based on their enhancement of targeting specificity. These include sequence conservation, strong base-pairing to the 3′ end of the miRNA, local AU content and location of miRNA binding sites (near either end of the 3′ UTR is favorable) (8). The importance of target structural accessibility for miRNA target recognition has been suggested by several studies (9–12). In particular, a target structure-based model validated with worm target data (12) was supported by an independent mammalian study with a higher true positive rate than the seed-based predictions (13). Findings from analyses of additional miRNA target data further support the importance of target structure (14–15).

While computational prediction algorithms have proven to be valuable in the discovery of new miRNA targets, current algorithms are known to make high numbers of false (positive or negative) predictions (16). In recent years, experimental methods based on crosslinking immunoprecipitation (CLIP) have been developed (7,17–21). CLIP methods involve UV irradiation of tissues, organisms or cells, for covalently crosslinking miRNA targets to the Argonaute (AGO) proteins, the catalytic components of the RISC complex. The crosslinked RNAs are reduced in size typically to ∼50 nt by partial RNase digestion. The short RNAs are amplified by RT-PCR and then sequenced for the identification of AGO tags that contain miRNA binding sites on target mRNAs. For mammalian systems, these include HITS-CLIP for mouse brain (18), PAR-CLIP for human cell lines (17), and variations referred to here for convenience as V-CLIP, V-PAR-CLIP and V-PAR-CLIP-MNase for the same cell lines as PAR-CLIP (20). Unlike previous experimental techniques of mRNA expression profiling and proteomics (22–24), these CLIP techniques allow direct identification of a huge pool of short target sequences that contain native miRNA binding sites. CLIP methods not only provide much higher resolution data with respect to the precise locations of the binding sites, but also avoid the problem of potential secondary effects associated with mRNA expression profiling and proteomics approaches. In addition, these CLIP datasets are powerful for revealing the presence of ‘seedless’ sites (non-canonical sites) and possible seed or seedless sites located in the coding sequences (CDS) or 5′ UTRs (4,25,26).

The CLIP studies have provided high throughout quality datasets for the identification of important miRNA binding site features as the basis for the development of a novel framework for improved predictions of miRNA binding sites. To pursue this objective, we started with a comprehensive enrichment analysis of target site features for both seed and seedless sites in all three mRNA regions (5′ UTR, CDS, 3′ UTR). Enriched miRNA binding site features were used for the development of logistic models for miRNA binding site predictions. Model-based predictions were evaluated by intra-dataset and inter-dataset validation, with the latter including cross-species validation. In addition, the predictions were compared with available predictions by several well-established algorithms.

## MATERIALS AND METHODS

**Figure 1.**
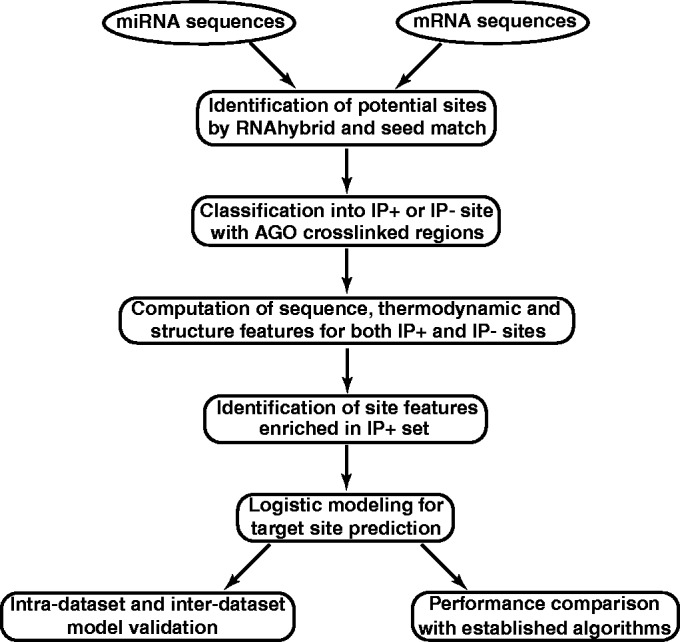
Flow chart for the CLIP data analysis and modeling.

The pipeline of CLIP data analysis and modeling is outlined in Figure 1, followed by description of procedures for each step of the pipeline.

### Description and processing of CLIP data

HITS-CLIP (18). For seed sites, we directly downloaded target sites identified by the authors (http://ago.rockefeller.edu/tag_mm9.php) for defining IP+ sites. For seedless sites, the raw AGO-mRNA HITS-CLIP Solexa sequencing data were downloaded from the authors’ website (http://ago.rockefeller.edu/rawdata.php) and then mapped to mouse genome using RMAP (27). A maximum of two mismatched nucleotides were allowed to ensure >90% identity in sequence alignment. Only reads with unique mapping were considered. For ensuring experimental reproducibility, two different antibodies, 2A8 with three biological replicates and 7G1-1 with two replicates, were used in the HITS-CLIP study. We identified the common tags reproduced by at least one replicate for each of the two antibodies. Two tags for the two antibodies are considered a common tag if and only if they have exactly the same nucleotide sequence and chromosomal location. The locations of the tags within target mRNAs were obtained with annotation from the Map Viewer of NCBI Build 37.2 for *Mus musculus* genome. Finally, we identified 6,666 unique AGO tags distributed across mRNAs of 2211 genes among 12 389 genes in P13 mouse brain used by the HITS-CLIP study (gene list provided by the authors). Thus, these 6666 AGO tags were used for our analysis. Among them, ∼61% tags were mapped to 3′ UTRs, ∼37% to CDS and ∼2% to 5′ UTRs. Each of these AGO tags is considered to harbor at least one miRNA binding site. The HITS-CLIP study focused on 20 most abundant miRNA families (114 miRNAs) comprising 88% of all AGO-associated miRNAs. We thus focused on these miRNA families in our data analysis and modeling.

PAR-CLIP (17). This study identified 17 319 miRNA site-containing AGO crosslink-centered regions (CCRs). Among exonic CCRs representing 85% of all AGO CCRs, 4% mapped to 5′ UTRs, 50% to CDS and 46% to 3′ UTRs. The 100 most abundant miRNAs in HEK 293 cells accounted for 95% of total miRNA sequence reads. These miRNAs were used in data analysis by the authors, and were the focus of our analysis. The AGO CCRs data were obtained from the authors. Based on genomic locations according to the annotation of *Homo sapiens* hg18, the CCRs are distributed across 5307 mRNAs among 22 466 transcripts expressed in human embryonic kidney (HEK) 293 cells (transcript list provided by the authors).

V-CLIP, V-PAR-CLIP, V-PAR-CLIP-MNase (20). We downloaded the three CLIP datasets from Gene Expression Omnibus: GSE28865. We selected experimental replicate A for our analysis due to higher reliability as reported (20). For V-CLIP, there are 54 905 CCRs distributed across 8818 mRNAs among 17 928 transcripts expressed in human HEK 293 cells. For V-PAR-CLIP, 91 362 CCRs are distributed across 9676 of 16 491 expressed transcripts. V-PAR-CLIP-MNase generated 44 497 CCRs across 8562 of 20 424 expressed transcripts. For each set of expressed transcripts, by using accession number, we compiled the list of those that are annotated in NCBI RefSeq database (build 36.3). This resulted in 44 557 CCRs across 7507 of 12 440 RefSeq transcripts for V-CLIP, 73 551 CCRs across 8099 of 11 660 transcript for V-PAR-CLIP and 37 125 CCRs across 7293 of 13 419 transcripts for V-PAR-CLIP-MNase. For V-CLIP, ∼2% of CCRs were mapped to 5′ UTRs, ∼31% to CDS and ∼67% to 3′ UTRs. For V-PAR-CLIP, ∼2% of CCRs were mapped to 5′ UTRs, ∼35% to CDS and ∼63% to 3′ UTRs. For V-PAR-CLIP-MNase, ∼3% of CCRs were mapped to 5′UTRs, ∼41% to CDS and ∼56% to 3′ UTRs. A set of the 10 most abundant miRNA families containing 44 mature miRNAs was used by the authors for analyses of data generated by the 3 CLIP protocols. These miRNAs were used in our study, with sequences provided by the authors and names updated using miRBase Release 18 (28).

### Identification of miRNA binding sites

To identify the potential miRNA binding sites, we used the RNAhybrid program (29). RNAhybrid was run using two options for aligning a miRNA with an mRNA. For the first option, as in our earlier work for worm (30), we used a threshold of −15 kcal/mol for ΔG_hybrid_ (a measure of hybrid stability) to identify potential sites with a stable hybrid and to account for potential seedless sites that can include rare centered sites (31). The second option is to force base-pairing for the seed region (miRNA nt 2-7, 2-8, 3-8) without enforcing an energy threshold. The latter will capture all cases of offset 6mer, 6mer, 7mer-A1, 7mer-m8 and 8mer seed sites (8). Some of these sites can have weak or no base-pairing beyond the seed region such that they would be missed by the first option due to the enforced energy threshold. However, the use of the two options can lead to site redundancy, e.g., two heavily overlapped sites of different seed types. To remove redundancy, we used the following procedure for selecting one out of multiple overlapped sites: 8mer is the most preferred, followed by 7mer-m8, 7mer-A1, 6mer, offset 6mer and seedless sites; in the case of same site types, the one with lower ΔG_hybrid_ (i.e., greater hybrid stability) is preferred. The procedure was based on the established seed hierarchy (8) and thermodynamic consideration.

### Site sequence features

To study the contribution of sequence characteristics on target recognition, we considered site sequence features as previously reported (8). These include seed types of offset 6mer, 6mer, 7mer-A1, 7mer-m8 and 8mer, miRNA 3′ compensatory/supplementary base-pairing as defined by the presence of contiguous WC base-pairing for miRNA nucleotide position 12-17, site location as defined by the location of the site relative to the length of the region (3′ UTR, CDS or 5′ UTR; e.g., for 3′ UTR, 0 indicates 5′ end of the 3′ UTR and 1 corresponds to the 3′ end.), AU content defined as the percentage of the adenosine or uridine bases for a nucleotide block either upstream or downstream of the site. The sizes of the block used for computing are 5, 10, 15, 20, 25 and 30 nt.

### Thermodynamic and target structure features

We computed ΔG_nucl_, a measure of nucleation potential, and ΔG_total_, a measure of total energy change for miRNA–target hybridization. These target structure-based parameters are key characteristics of our two-step model for miRNA:target interaction (12). ΔG_hybrid_, a measure of the stability of miRNA–target duplex as computed by RNAhybrid (29) was also included in our analysis. While ΔG_total_ is an energetic measure of structural accessibility at the target site, we also computed several probabilistic measures of structural accessibility as follows. For a block of nucleotides, the accessibility can be measured by the average probability that a nucleotide in the block is single stranded, based on the RNA secondary structure sampling algorithm implemented by Sfold (32,33). We considered the entire target site for computing site accessibility, the block within the target site complementary to miRNA seed for seed accessibility, and the block upstream or downstream the target site for upstream accessibility or downstream accessibility. The sizes for upstream or downstream block are 5, 10, 15, 20, 25 and 30 nt.

### Conservation score

We calculated conservation score for each binding site. For seed sites, we also computed the conservation score for the seed complementary region within the target site, and for the set of target site nucleotides outside the seed complementary region (off-seed region). The conservation score for a set of nucleotides is the average of conservation scores for individual nucleotides. The nucleotide conservation score files were downloaded from UCSC genome browser (http://genome.ucsc.edu/). These files were generated by the PhastCons program (35) through multiple-sequence alignments of 44 vertebrate genomes to the human genome (hg18) and 30 vertebrate genomes to the mouse genome (mm9). The hg18 and mm9 track tables in UCSC genome browser were used to map the nucleotide conservation scores from chromosomes to mRNAs.

### Classification of binding sites into IP+ set and IP− set

Among potential miRNA binding sites (seed or seedless) predicted by RNAhybrid, those reside within an AGO-crosslinked region (tag or CCR) were considered IP+ sites. The remaining sites define the set of IP− sites.

### Enrichment analysis

Seed types and miRNA 3′ compensatory/supplementary base-pairing are categorical features, whereas others are continuous. The objective is to identify the categories or intervals of the features that are enriched for the IP+ set. For conservation score, some sites (<1% of all sites) were removed because their conservation scores were not available due to the absence of their mRNAs in the UCSC track tables. Given a category (e.g., 8mer) or an interval (e.g., (−10 kcal/mol, −9 kcal/mol) for ΔG_total_, (0.4, 0.5) for conservation score), we measured the degree of enrichment by the ratio of the odds of the feature category or interval occurring in the IP+ set to the odds of it occurring in IP− set. The odds are *P*/(1 − *P*), with *P* estimated by the frequency (percentage) of the feature category or interval in the whole IP+ or IP− set. If the enrichment (odds) ratio = 1, the feature category or interval is equally likely to occur in the IP+ set and the IP− set; ratio > 1 indicates more likely occurrence in the IP+ set and thus enrichment of the feature; ratio < 1 indicates more likely occurrence in the IP− set and depletion of the feature.

### Logistic regression modeling for prediction of miRNA binding sites

For model-based prediction of miRNA binding sites, we employed logistic regression (35) to address the problem of predicting binary outcome. For potential target sites, the two possible outcomes are 1 for IP+ and 0 for IP−. Because the enrichment of a feature in the IP+ set indicates its predictive value, we included all enriched features in the development of multivariate logistic regression model using established procedures (35) and the R package (http://www.r-project.org), an open source statistical software package. For categorical features, we used the odds ratio of each category to represent their values for sites belonging to this category. We focused on non-linear logistic modeling, as some of the features may be correlated, e.g., AU content is correlated to structural accessibility (8). For the vector of enriched features, *X* = {*X_i_*}, representing a binding site, the non-linear logistic model was defined as log[*P*(*X*)/(1 − *P*(*X*)] = *α* + ∑*β_i_ X_i_* + ∑ *_i_*_≠_*_j_* γ*_ij_ X_i_ X_j_*, where *X_i_ X_j_* is an interaction (i.e., quadratic) term which measures potential interaction of a pair of likely correlated features. The logistic model was trained on each CLIP dataset. For modeling incorporating conservation score, we excluded sites for which conservation scores were not available. The IP− set is larger than the IP+ set by ≥∼7-fold for seed sites, and by at least an order of magnitude for seedless sites. To avoid potential bias in model fitting due to imbalance in the sizes of datasets, we randomly selected a subset of the IP− set with its size equal to that of the IP+ set. We then trained a logistic model on the whole IP+ set and the selected IP− subset. The random selection from the IP− subset and the subsequent model training were performed 10 times. For a model-testing site with feature vector *X^t^* = {*X^t^_i_*}, logistic model fitting returns a classification probability for each of the 10 iterations. We used *P*(*X^t^*), the average of the probabilities of the 10 iterations as a single probabilistic prediction that the site is a binding site. Thus, for a pre-specified probability threshold *q*, a deterministic prediction can be made, i.e., the testing site is predicted to be a binding site if and only if *P*(*X^t^*) ≥ *q*.

### Model performance assessment

The true positive rate (TPR = sensitivity), false positive rate (FPR = 1 − specificity) are commonly used measures for assessing the performance of a prediction model. By changing probability threshold *q,* we constructed receiver operator characteristic (ROC) curve plotting the TPR against the FPR. However, neither TPR nor FPR provides an overall measure of performance. By changing *q*, sensitivity can be improved at the cost of decreased specificity and vise versa. For an alternative quantitative assessment of performance, we used the Youden’s J statistic (Youden’s index) (36). The Youden’s J statistic is an overall measure of performance defined as (TPR − FPR) = sensitivity + specificity − 1.

For applications of the models, the top-ranked predictions are of high interest for experimental testing, as they are expected to have a high likelihood of positive validation. We thus also computed the TPR for top-ranked predictions. For a given set of top-ranked sites predicted by a method, the TPR is computed by the number of true positive sites (according to CLIP data) divided by the total number of the top-ranked sites.

### Intra-dataset validation

For each CLIP dataset, intra-dataset validation was performed with the standard 10-fold cross-validation strategy, to avoid the potential problem of model over-fitting. The IP+ set and the IP− set were each randomly divided into 10 subsets of equal size. At the *i*-th repetition (*I* = 1, … ,10), the *i-*th IP+ subset and the *i-*th IP− subset were selected for testing of model accuracy, the remaining nine IP+ subsets were combined into set *A^i^*, a subset of the entire IP+ set, and the remaining nine IP− subsets were combined into set *B^i^*, a subset of the entire IP− set. These two datasets were used for model training. The problem of data imbalance between *A^i^* and *B^i^* is addressed by the strategy described for logistic regression modeling above. The final modeling performance was then computed by averaging over the 10 repetitions.

### Inter-dataset validation

The availability of multiple datasets generated by similar experimental protocols presents the opportunity for model validation by independent data (inter-dataset validation). We performed inter-dataset validation using the five CLIP datasets by training logistic model on IP+ and IP− sets from one CLIP dataset then testing model performance on the other four CLIP datasets. For model training, data imbalance between IP+ and IP− sets was also addressed.

### Performance comparison with other algorithms

For an established algorithm to be included in our performance comparison, either target site predictions are available for mm9 and hg18 or hg19, or the software implementing the algorithm can be run locally to generate predictions. To this end, we identified four target prediction algorithms: the sequence-based TargetScan (37), the structure-based PITA (11), the composite-feature-based mirSVR (38) and the pattern-based RNA22 (39). TargetScan 6.0 was downloaded from (http://www.targetscan.org/) and run locally with default setting. PITA version 6 was downloaded from (http://genie.weizmann.ac.il/pubs/mir07/mir07_exe.html) and run locally with the settings: one GU, one mismatch in seed region. mirSVR database (August 2010 Release) was downloaded from (http://www.microrna.org). Because hg19 was the newest version for mirSVR prediction database, we further processed mirSVR predictions to ensure that all of the mirSVR binding sites can be found in hg18 for comparison with predictions for hg18. RNA22 database (miRNAs from miRBase 16 and genes from ENSEMBL 62) was downloaded from (http://cm.jefferson.edu/rna22v1.0/). Same as mirSVR, we also further processed RNA22 prediction for comparison with predictions for hg18. For seed sites in 3′ UTRs, predictions by the four algorithms were compared with our CLIP-based logistic models. For seedless sites, TargetScan does not make predictions, and PITA, mirSVR and RNA22 prefer to predict the class with one GU pair and/or one mismatch in the seed complementary region. We thus considered this class of seedless sites for performance comparison.

For construction of a ROC curve by varying the threshold for a score, we used context score for TargetScan, mirSVR score for mirSVR, structure-based energy score for PITA, and pattern-based score for RNA22. For overall performance comparison, we present the highest possible value of Youden’s J statistic by identifying the threshold for the score of each of these algorithms that maximizes the difference between TPR and FPR. We note that the scores of these algorithms are incompatible with logistics probability, so that the comparison using Youden’s J statistic is limited to the best-performing score threshold for each of these algorithms.

## RESULTS

### Identification of enriched target site features

For each of the sequence, thermodynamic and structure features, we performed enrichment analysis. For both seed and seedless sites, the results are highly similar for HITS-CLIP, PAR-CLIP, V-CLIP and V-PAR-CLIP, for which more enriched features were observed than for V-PAR-CLIP-MNase (Supplementary Tables S1–S5), suggesting a substantially different profile by MNase for the AGO CCRs. The enrichment signals for V-CLIP are appreciably stronger than HITS-CLIP, PAR-CLIP and V-PAR-CLIP. Among the three regions of mRNAs, the enrichment signals are strongest for 3′ UTRs and weakest for 5′ UTRs. Below we summarize our findings on 3′ UTRs for V-CLIP data.

**Figure 2.**
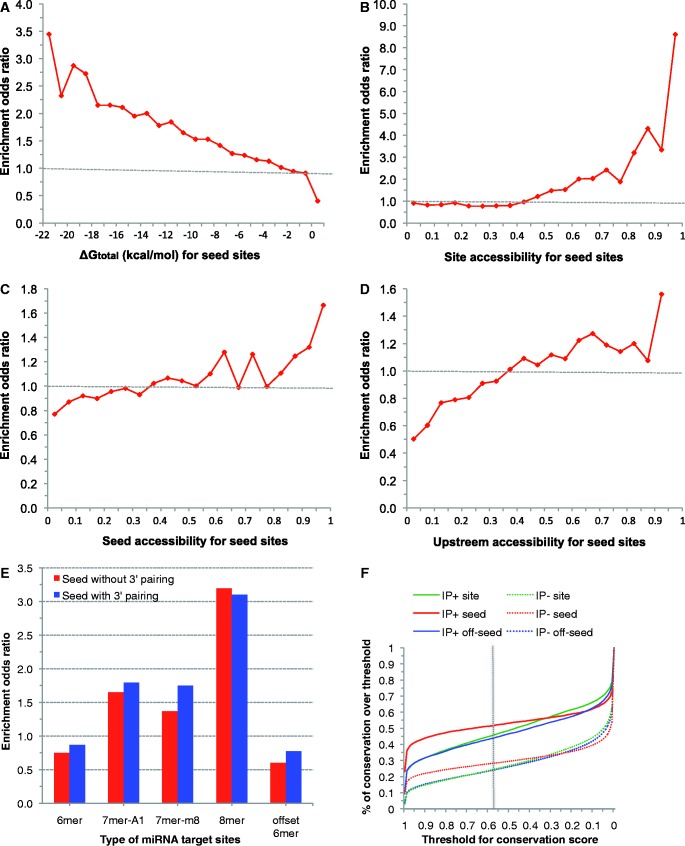
Enrichment of representative seed site features (3′ UTRs for V-CLIP data): (**A**) ΔG_total_; (**B**) site accessibility; (**C**) seed accessibility; (**D**) upstream accessibility (window size of 15 nt); (**E**) type of miRNA target sites; (**F**) percentage (*Y*-axis) of sites/seed/off-seed regions with conservation scores greater than or equal to a pre-specified threshold (*X*-axis), in the IP+ set or the IP− set (dashed line corresponding to a threshold of 0.57 previously used for defining conservation (34,38).

Among all of the seed sites, only 7% are within the AGO CCRs (i.e., IP+) from the V-CLIP study (12% for 8mer and 7mer sites), indicating that seed alone is a predictor with high FPR. Features enriched for IP+ seed sites include Δ*G*_nucl_, Δ*G*_total_ (Figure 2A), site accessibility (Figure 2B), seed accessibility (Figure 2C), upstream accessibility (window size of 15 nt, Figure 2D), downstream accessibility (10 nt), 8mer and 7mer seed, 3′ base-pairing, upstream AU (30 nt), downstream AU (30 nt), site location, seed conservation and site conservation. For example, more favorable energy is enriched for Δ*G*_total_. In particular, Δ*G*_total_ is the only feature enriched for all three mRNA regions, for both seed and seedless sites, and for all five CLIP datasets. Among enriched 8mer, 7mer-A1 and 7mer-m8, the enrichment of 8mer is the strongest. The enrichment of 7mer-A1 and 7mer-m8 are enhanced by 3′ base-pairing (Figure 2E), consistent with the supplementary role of 3′ base-pairing (8). However, 3′ base-pairing does not enhance the enrichment for 8mer seed sites. There is a lack of enrichment for 6mer and offset 6mer seed sites. There is a far greater conservation for IP+ seed sites, with higher conservation for seed complementary region than the whole target site (Figure 2F). For the 3′ UTR, the gap in conservation score between IP+ and IP− is larger than that of CDS or 5′ UTR. These suggest that miRNA bindings sites in particular the seed complementary regions tend to reside in conserved blocks on target 3′ UTR. For the threshold of 0.57 (vertical dashed line in Figure 2F) for indicating conservation across all mammals (34), the seed complementary region is conserved for ∼52% in IP+ seed sites, but only ∼28% in IP− seed sites, further supporting the importance of seed.

**Figure 3.**
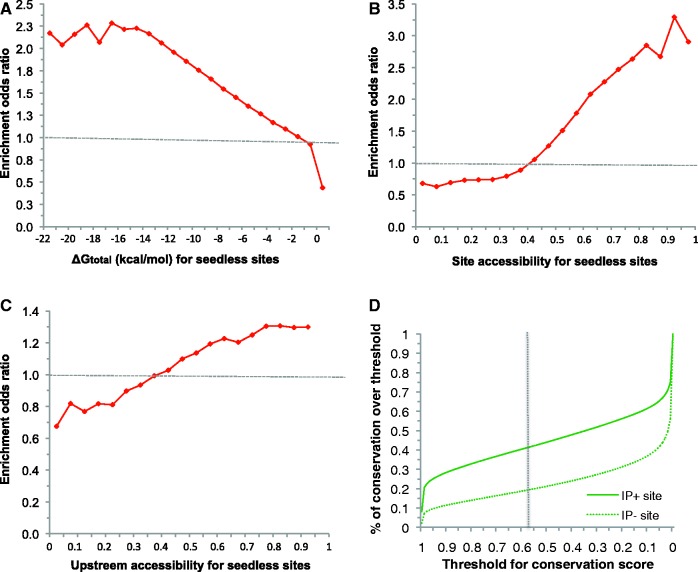
Enrichment of representative seedless site features (3′ UTRs for V-CLIP data): (**A**) ΔG_total_; (**B**) site accessibility; (**C**) upstream accessibility (window size of 10 nt); (**D**) percentage (*Y*-axis) of seedless sites with conservation scores greater than or equal to a pre-specified threshold (*X*-axis), in the IP+ set or the IP− set.

Among all of the identified miRNA binding sites residing within the AGO CCRs, ∼96% are seedless. This together with conservation evidence (Figure 3D) strongly suggests substantial involvement of seedless sites in gene regulation by miRNAs. For seedless sites and for features that are not seed related, the results of enrichment analysis are similar to those for seed sites (Supplementary Tables S1–S5). The enriched features include ΔG_nucl_, ΔG_total_ (Figure 3A), site accessibility (Figure 3B), upstream accessibility (10 nt, Figure 3C), downstream accessibility (30 nt), 3′ base-pairing, upstream AU (30 nt), downstream AU (30 nt), site location and site conservation (Figure 3D).

### Prediction of seed sites in 3′ UTRs and performance evaluation

**Figure 4.**
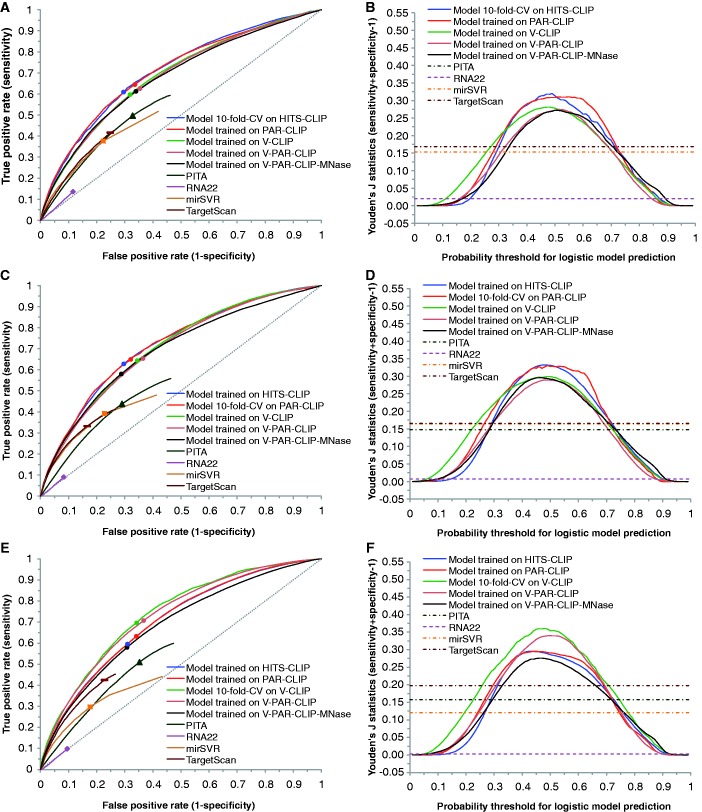

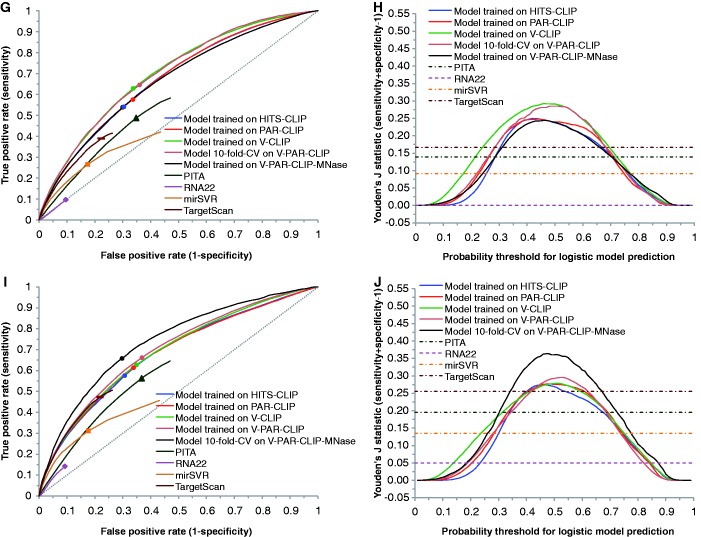
Inter-dataset model validation by using one of the five CLIP datasets as an independent set for testing performance of each of models developed with other four datasets; intra-dataset validation by 10-fold cross validation (CV); and performance comparison with established algorithms for predictions of seed sites in 3′ UTRs (dashed diagonal line for random predictions). ROC curve and Youden’s J statistic are shown for model testing on HITS-CLIP data (**A**, **B**), on PAR-CLIP data (**C**, **D**), on V-CLIP data (**E**, **F**), on V-PAR-CLIP data (**G**, **H**) and on V-PAR-CLIP-MNase data (**I**, **J**). The color-matched dots on ROC curves correspond to a logistic probability threshold of 0.5. The rectangle, triangle, square and diamond correspond to the best-performing score threshold (according to Youden’s J statistic) for TargetScan, PITA, mirSVR and RNA22, respectively.

For each CLIP dataset, using enriched features identified for the dataset, we first performed logistic model training. The model performance was tested by both intra-dataset validation and inter-dataset validation. For seed sites in 3′ UTRs, we compared our predictions with predictions available from TargetScan (37), mirSVR (38), PITA (11) and RNA22 (39). For a wide range of logistic probability threshold centering around 0.5, logistic models trained on the five CLIP datasets all have substantially higher TPR than TargetScan and mirSVR, RNA22 with comparable FPR, and lower FPR than PITA, RNA22 with comparable TPR (Figure 4A, C, E, G and I). Furthermore, there are major overall improvements as measured by the Youden’s J statistic (Figure 4B, D, F, H and J). Among the four established algorithms, TargetScan is the best performer, followed by PITA, mirSVR and RNA22.

We also computed the TPR of top-ranked (from top 1% to 10%) predictions for each of the five CLIP datasets (Supplementary Figure S1a, c, e, g and i). Our logistic model generally outperforms TargetScan and mirSVR, and is substantially better than PITA and RNA22, especially for more highly ranked predictions (e.g., 1%).

For inter-dataset validation, the model trained on V-PAR-CLIP-MNase is the worst performer among the five CLIP-based logistic models, further indicating substantial difference between the data for MNase and the data for other CLIP protocols. For model testing on the HITS-CLIP dataset from mouse brain (Figure 4A and B), logistic models trained on HITS-CLIP and PAR-CLIP are the two best performers. For logistic probability threshold of 0.5, the improvement by either model in Youden’s J statistic is about 0.13 over TargetScan and PITA, 0.15 over mirSVR and 0.28 over RNA22. For each of the remaining three models trained on V-CLIP, V-PAR-CLIP, V-PAR-CLIP-MNase datasets, the improvement is about 0.1 over TargetScan and PITA, 0.12 over mirSVR and 0.25 over RNA22.

For cross-species validation, we observed good results on mouse CLIP data for models trained on human CLIP datasets (Figure 4A and B). Furthermore, the HITS-CLIP-based model is well validated on all human CLIP datasets (Figure 4C–J). In particular, for testing performance on any of the human CLIP datasets, the HITS-CLIP-based model performed as well as the PAR-CLIP-based model. These findings suggest that our analysis captured intrinsically important target site characteristics that are species independent. Thus, the model developed for one species using quality CLIP data may be generally applicable to target site prediction for other mammalian species and beyond, because the regulatory machineries are either identical or highly similar.

For each of the four models trained on different human CLIP datasets, we computed the average performance of both intra-dataset and inter-dataset validation using all four human CLIP datasets. This analysis showed that V-CLIP derived model is the best performer (Supplementary Figure S2a and b). The improved predictability by V-CLIP model is an expected result due to stronger enrichment signals for V-CLIP data. The findings from enrichment analysis and model comparison suggest that data from V-CLIP has the best quality, at least from the perspective of data analysis and modeling.

### Prediction of seedless sites

**Figure 5.**
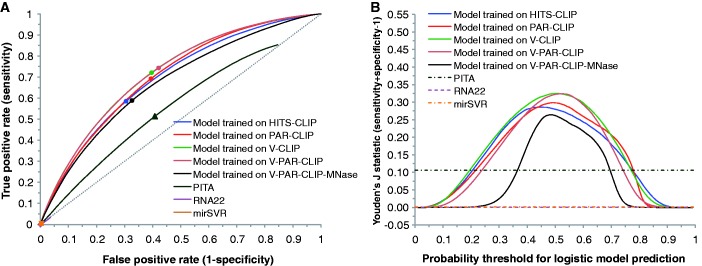
Performance comparison of logistic models trained on five CLIP data sets with established algorithms for predictions of seedless sites with one GU pair and/or one mismatch within seed complementary region in 3′ UTRs (dashed diagonal line for random predictions). ROC curve (**A**) and Youden’s J statistic (**B**) are shown for model testing on V-CLIP.

The seedless site predictions are similar to seed site predictions in the trends of ROC curves and Youden’s J statistic, as well as in the levels of improvements over random predictions (Supplementary Figure S3). For seedless sites with one GU pair and/or one mismatch in the seed complementary region, a class of seedless sites predicted by all of PITA, mirSVR and RNA22, we found that levels of improvements by logistic modeling are greater than those for seed site predictions (Figures 4 and 5; Supplementary Figure S4). We note that the TPRs and FPRs for mirSVR and RNA22 are very low, due to the small number of predictions for this class of seedless sites that are available from their databases. Again, V-CLIP derived model is the best performer among four models derived from human CLIP datasets (Supplementary Figure S2c–f). For top-ranked predictions, our model has a substantially higher TPR than mirSVR, PITA and RNA22 (Supplementary Figure S1b, d, f, h and j).

### Prediction for both CDS and 5′ UTR

The enriched features identified for CDS and 5′ UTRs allowed us to develop models specifically for these two regions. For both seed and seedless sites, the predictions are similar to those for 3′ UTRs in the trends of ROC curves and Youden’s J statistic, as well as in the levels of improvements over random predictions (Supplementary Figure S5). Again, V-CLIP derived model is the best performer among four models for human CLIP datasets (Supplementary Figure S6).

### Software and database availability

We have implemented the HITS-CLIP-based logistics regression model for mouse and the V-CLIP-based model for human into the STarMir module of the Sfold software (http://sfold.wadsworth.org/cgi-bin/starmir.pl), allowing users to submit any miRNA and mRNA for prediction of binding sites by our models. We have also developed STarMirDB (http://sfold.wadsworth.org/starmirDB.php), a web searchable database currently including all predicted binding sites by our models for miRNAs and mRNAs in the HITS-CLIP and V-CLIP studies, with an indicator showing whether a site is supported by the CLIP study. The database is being extended to include whole transcriptome predictions for human and mouse.

## DISCUSSION

The high throughput datasets from CLIP studies presented a unique opportunity for us to identify target site characteristics important for binding by miRNAs, to develop novel statistical models for target site prediction, and to test performance of models by not only the conventional intra-dataset validation, but also inter-dataset validation including cross-species validation. The major improvement in performance by our approach over established algorithms is partly attributable to the high quality of the data from the CLIP studies.

From the comprehensive enrichment analyses, we found that data from the CLIP studies not only support the importance of most target site features reported in the literature, but also suggest several modifications and reveal previously unknown characteristics. Importantly, most of the findings are common not only for both seed and seedless sites but also for all of three regions of mRNA.

Although three types of seed sites, 8mer, 7mer-m8 and 7mer-A1, were found to be enriched in IP+ set, the levels of enrichment for 7mer-m8 and 7mer-A1 are highly comparable (Figure 2E). Thus, there is a lack of support for greater effectiveness by 7mer-m8 over 7mer-A1 as previously reported (8). In contrast to 7mer-m8 and 7mer-A1, presence of 3′ base-pairing may not enhance enrichment for 8mers (Figure 2E), perhaps due to adequate stability for the seed portion of the miRNA:target hybrid for 8mers.

Our consideration of seedless sites in addition to seed sites was supported by evolutionary conservation (Figure 3D). Although the CDS is much more conserved than the 3′ UTR, the signal of enrichment for CDS was weaker than 3′ UTR, perhaps due to the possibility that greater portion of CLIP hits for CDS are for transient rather than stable association between RNAs and AGO. Although it is impossible to distinguish transient binding from stable binding, binding can still be predicted for each region using a region-specific model. Furthermore, the seed complementary region is more conserved than the remaining nucleotides within the entire seed site (Figure 2F), further supporting the significance of conserved seed pairing (3).

There is a lack of enrichment for ΔG_hybrid_ with the exception of data for seed sites from V-PAR-CLIP-MNase (Supplementary Tables S1–S5). This indicates that miRNA:target hybrid stability is generally not important for target recognition, and further suggests the CLIP data profile for MNase is different from those for other studies. We found that Δ*G*_total_ is the only feature enriched for all CLIP datasets, all three target regions, and for both seed and seedless sites. Thus, as one of the important factors for target binding by miRNAs in general, target structural accessibility is likely the most important factor for seedless sites. The findings lend additional support to the structure-based hybridization model with Δ*G*_total_ as the key energetic characteristic (12). For region upstream or downstream of target site, we observed enrichment of accessibility as measured via structure prediction or by AU content. This is consistent with the report that the RISC complex can bind non-specifically to single-stranded regions near target sites to facilitate target recognition (40).

A previous analysis of target site features examined 3′ UTRs and a subset of abundant miRNAs from HITS-CLIP and PAR-CLIP studies (41). In their study, only the single best predicted site on a 3′ UTR was considered, and seed sites and seedless sites were not separated. Different methods were used for the computation of some of the features and for the analysis and modeling of CLIP data. Despite the differences in scope and methodology for analysis and modeling, some of the important features reported in their study (e.g., conservation and accessibility of target site, upstream or downstream AU content) are further supported by our enrichment analysis.

In comparison to other established algorithms, our analysis and modeling framework offers much greater flexibility in selection of enriched features for different CLIP datasets, in modeling the contribution of each enriched feature, and in modeling potential interactions between different features. Furthermore, our approach makes predictions for broad classes seedless sites including sites with one GU or one mismatch for the seed complementary region, G-bulge sites (42), sites with 3′ complementary base-pairing (8), centered sites (31) and beyond. Such capability will facilitate studies on widespread targeting by seedless sites (7).

We chose the nonlinear option of logistic regression for model development. This addresses potential correlation between some of the enriched features. For example, AU content and the probabilistic measure of structural accessibility could be correlated, as AU-rich regions are more likely to be single stranded and thus structurally accessible. We also performed linear logistic modeling. We found that, in general, the nonlinear option outperformed the linear option (Supplementary Figure S7). Our approach presents a probability from logistic regression modeling for predicting a miRNA binding site. Sites with higher probabilities will have a greater chance for positive experimental validation (see Supplementary Figure S1).

The similar results by enrichment analysis and the generally good performances from inter-dataset validations indicate that our modeling framework is robust across independent datasets generated by different experimental protocols for different species. The findings also indicate that majority of characteristics for miRNA binding sites are shared by different species, because the regulatory machineries are either identical or highly similar. These suggest that a model trained on data from a high throughput quality CLIP study for one species may be applicable to binding site predictions for another species.

The findings from our study confirm the power of CLIP data for the development of improved algorithm for prediction of miRNA binding sites. However, the CLIP techniques and available CLIP data have a number of limitations. The identity of the miRNA(s) binding to an AGO crosslinked regions is unknown and needs to be predicted for further analysis. The abundant miRNAs in these studies only represent a small portion of all known human or mouse miRNAs. The majority of the mRNAs in the complete human or mouse transcriptome are absent in the CLIP datasets, due to either lack of expression or a low level of expression. Furthermore, CLIP experiments are difficult to perform, and successful applications are still limited. For example, successful CLIP application has not been reported for fly. Therefore, our prediction models will greatly complement the existing CLIP data by enabling genome-scale miRNA target predictions for any species of interest with higher prediction accuracy than the established algorithms.

The CLIP methods are binding assays rather than functional assays. Therefore, models developed from CLIP data are limited to prediction of miRNA binding sites. In addition, the best large-scale data on miRNA binding sites are from CLIP studies. For these reasons, we considered model validation by independent CLIP data (i.e., inter-dataset validation) as the gold standard for validation and performance comparison. These models do not make functional predictions that include the outcome of miRNA binding (i.e., target degradation or translational repression) and the degree of regulation on either the mRNA or the protein level. Such predictions will require computational modeling of the effects of multiple miRNA binding sites on the same target, as well as analysis and modeling of high throughput functional data for miRNA:target interactions.

## SUPPLEMENTARY DATA

Supplementary Data are available at NAR Online: Supplementary Tables 1–5, Supplementary Figures 1–7 and Supplementary Text File.

## FUNDING

National Science Foundation (NSF) [DBI-0650991 to Y.D.]; National Institutes of Health (NIH) [GM099811 to Y.D.]; Nafosted Fund of Vietnam [102.03-2010.04 to D.L.]. Funding for open access charge: NSF and NIH grants.

*Conflict of interest statement*. None declared.

## Supplementary Material

Supplementary Data
