# Supplementary material for: CLIP-based prediction of mammalian microRNA binding sites
Source: Nucleic Acids Res. 2013 May 22;41(14):e138. doi: 10.1093/nar/gkt435 (PMC3737542; doi:10.1093/nar/gkt435)
Supplement: Supplementary Data [file supp_41_14_e138__index.html]

CLIP-based prediction of mammalian microRNA binding sites — Supplementary Data 

# CLIP-based prediction of mammalian microRNA binding sites

## Supplementary Data

files

**Files in this Data Supplement:**

- Supplementary Data - pdf file
