# Supplementary material for: CLIP-based prediction of mammalian microRNA binding sites
Source: Nucleic Acids Res. 2013 May 22;41(14):e138. doi: 10.1093/nar/gkt435 (PMC3737542; doi:10.1093/nar/gkt435)
Supplement: Supplementary Data [file supp_gkt435_Supplementary_all.pdf]

CLIP-Based Prediction of Mammalian MicroRNA Binding Sites

Chaochun Liu, Bibekanand Mallick, Dang Long, William A. Rennie,
Adam Wolenc, C. Steven Carmack and Ye Ding

List of Supplementary Tables and Figures

**Supplementary Table 1**	Summary of feature enrichment analysis for HITS-CLIP data
**Supplementary Table 2**	Summary of feature enrichment analysis for PAR-CLIP data
**Supplementary Table 3**	Summary of feature enrichment analysis for V-CLIP data
**Supplementary Table 4**	Summary of feature enrichment analysis for V-PAR-CLIP data
**Supplementary Table 5**	Summary of feature enrichment analysis for V-PAR-CLIP-MNase data
**Supplementary Figure 1**	True positive rate comparison for top ranked predictions of seed sites and seedless sites with one GU pair and/or one mismatch in the seed complementary region in 3′ UTRs
**Supplementary Figure 2**	Average performance evaluation for each of four models trained on human CLIP data by testing on all four human CLIP datasets (i.e., one intra-dataset and three inter-dataset validations) for seed sites, all seedless sites and seedless sites with one GU pair and/or one mismatch within seed complementary region in 3′ UTRs
**Supplementary Figure 3**	Intra-dataset and inter-dataset validation of five CLIP-based models (seedless sites in 3′ UTRs)
**Supplementary Figure 4**	Intra-dataset and inter-dataset validation of five CLIP-based models (seedless sites with one GU pair and/or one mismatch within seed complementary region in 3′ UTRs)
**Supplementary Figure 5**	Intra-dataset and inter-dataset validation of five CLIP-based models (seed and seedless sites in CDS and 5′ UTRs)
**Supplementary Figure 6**	Average performance evaluation for each of four models trained on human CLIP data by testing on all four human CLIP datasets (i.e., one intra-dataset and three inter-dataset validations) for seed and seedless sites in CDS and 5′ UTRs
**Supplementary Figure 7**	Performance comparison between nonlinear logistic modeling and linear logistic modeling

Supplementary Figure Legends

Supplementary Figure 1. True positive rate comparison for top ranked (from top 1% to 10%) predictions of seed sites (left panel) and seedless sites with one GU pair and/or one mismatch in the seed complementary region (right panel) in 3′ UTRs for testing of models on HITS-CLIP data (**a**, **b**), on PAR-CLIP data (**c**, **d**), on V-CLIP data (**e**, **f**), on V-PAR-CLIP data (**g**, **h**), and on V-PAR-CLIP-MNase data (**i**, **j**).

Supplementary Figure 2. Average performance evaluation for each of four models trained on human CLIP data by testing on all four human CLIP datasets (i.e., one intra-dataset and three inter-dataset validations) and average performance comparison with established algorithms for predictions in 3′ UTRs (dashed diagonal line for random predictions). Average ROC curve and Youden's J statistic are shown in for seed sites (**a, b**), for all seedless sites (**c, d**), and for seedless sites with one GU pair and/or one mismatch within seed complementary region (**e, f**).

Supplementary Figure 3. Inter-dataset model validation by using one of the five CLIP datasets as an independent set for testing performance of each of models developed from other four datasets; intra-dataset validation by ten-fold cross validation (CV) (dashed diagonal line for random predictions) for seedless sites in 3′ UTRs. For each figure, ROC curve and Youden's J statistic are shown for model testing on HITS-CLIP data (**a, b**), on PAR-CLIP data (**c, d**), on V-CLIP data (**e, f**), on V-PAR-CLIP data (**g, h**), and on V-PAR-CLIP-MNase data (**i, j**).

Supplementary Figure 4. Performance comparison of logistic models trained on five CLIP datasets with PITA, RNA22 and mirSVR for prediction of seedless sites with one GU pair and/or one mismatch within seed complementary region in 3′UTRs. For each figure, ROC curve and Youden's J statistic are shown for model testing on HITS-CLIP data (**a, b**), on PAR-CLIP data (**c, d**), on V-PAR-CLIP data (**e, f**), and on V-PAR-CLIP-MNase data (**g, h**).

Supplementary Figure 5. Inter-dataset model validation by using one of the five CLIP datasets as an independent set for testing performance of each of models developed from other four datasets; intra-dataset validation by ten-fold cross validation (CV) (dashed diagonal line for random predictions) for seed sites in CDS (**5.1-10)**, seed sites in 5′ UTRs (**5.11-20**), seedless sites in CDS (**5.21-30**) and seedless sites in 5′ UTRs (**5.31-40**). For each figure, ROC curve and Youden's J statistic are shown for model testing on HITS-CLIP data (**5.1-2**, **5.11-12**, **5.21-22**, **5.31-32**), on PAR-CLIP data (**5.3-4**, **5.13-14**, **5.23-24**, **5.33-34**), on V-CLIP data (**5.5-6**, **5.15-16**, **5.25-26**, **5.35-36**), on V-PAR-CLIP data (**5.7-8**, **5.17-18**, **5.27-28**, **5.37-38**), and on V-PAR-CLIP-MNase data (**5.9-10**, **5.19-20**, **5.29-20**, **5.39-40**).

Supplementary Figure 6. Average performance evaluation for each of four models trained on human CLIP data by testing on all four human CLIP datasets (i.e., one intra-dataset and three inter-dataset validations) for seed sites in CDS, 5′ UTRs and seedless sites in CDS, 5′ UTRs (dashed diagonal line for random predictions). Average ROC curve and Youden's J statistic are shown for seed sites in CDS (**a, b**), seed sites in 5′ UTRs (**c, d**), seedless sites in CDS (**e, f**), seedless sites in 5′ UTRs (**g, h**).

Supplementary Figure 7. Performance comparison between nonlinear logistic modeling and linear logistic modeling. ROC curve and Youden's J statistic are shown for model tested on V-CLIP data for

seed sites in 3′ UTRs (**a, b**), in CDS (**c, d**), tested on HITS-CLIP for seedless sites in 3′ UTRs (**e, f**), in 5′UTRs (**g, h**).

Supplementary Table 1

Summary of feature enrichment Analysis for HITS-CLIP data

Features	Seed			Seedless		
	3′ UTR	CDS	5′ UTR	3′ UTR	CDS	5′ UTR
ΔG_{total}	•	•	•	•	•	•
ΔG_{hybrid}						
ΔG_{nucl}	•	•		•	•	•
Seed type	•	•	•			
miRNA 3′ base-pairing	•	•	•	•	•	•
Site accessibility	•	•	•	•	•	•
Seed accessibility	•	•				
Upstream accessibility	• 15 nt	• 15 nt		• 15 nt	• 15 nt	• 20 nt
Downstream accessibility	• 15 nt	• 15 nt		• 15 nt	• 15 nt	• 20 nt
Upstream AU content	• 30 nt	• 30 nt	• 20 nt	• 30 nt	• 30 nt	• 20 nt
Downstream AU content		• 30 nt			• 30 nt	• 30 nt
Site conservation score	•	•		•	•	•
Seed conservation score	•	•				
Site location	•	•		•	•	•

1. • indicates enrichment;
2. Number of nts gives the size of the nucleotide block for which the strongest enrichment was observed.

Supplementary Table 2

Summary of feature enrichment analysis for PAR-CLIP data

Features	Seed			Seedless		
	3′ UTR	CDS	5′ UTR	3′ UTR	CDS	5′ UTR
ΔG_{total}	•	•	•	•	•	•
ΔG_{hybrid}						
ΔG_{nucl}	•	•				•
Seed type	•	•	•			
miRNA 3′ base-pairing	•	•	•	•	•	•
Site accessibility	•	•	•	•	•	•
Seed accessibility		•				
Upstream accessibility		• 15 nt		• 10 nt	• 10 nt	• 10 nt
Downstream accessibility	• 10 nt	• 20 nt	• 10 nt	• 10 nt	• 10 nt	• 10 nt
Upstream AU content	• 30 nt	• 20 nt	• 30 nt	• 30 nt	• 30 nt	• 25 nt
Downstream AU content		• 25 nt	• 25 nt	• 15 nt	• 15 nt	• 30 nt
Site conservation score	•	•		•	•	•
Seed conservation score	•	•				
Site location	•		•	•		•

1. • indicates enrichment;
2. Number of nts gives the size of the nucleotide block for which the strongest enrichment was observed.

Supplementary Table 3

Summary of feature enrichment analysis for V-CLIP data

Features	Seed			Seedless		
	3′ UTR	CDS	5′ UTR	3′ UTR	CDS	5′ UTR
ΔG_{total}	•	•	•	•	•	•
ΔG_{hybrid}						
ΔG_{nucl}	•	•		•	•	•
Seed type	•	•	•			
miRNA 3′ base-pairing	•	•	•	•	•	•
Site accessibility	•	•	•	•	•	•
Seed accessibility	•	•				
Upstream accessibility	• 15 nt	• 30 nt		• 10 nt	• 10 nt	• 10 nt
Downstream accessibility	• 10 nt	• 30 nt	• 30 nt	• 30 nt	• 15 nt	• 10 nt
Upstream AU content	• 30 nt	• 20 nt	• 30 nt	• 30 nt	• 20 nt	• 25 nt
Downstream AU content	• 30 nt	• 30 nt	• 20 nt	• 30 nt	• 20 nt	• 30 nt
Site conservation score	•	•		•	•	•
Seed conservation score	•	•				
Site location	•		•	•	•	•

1. • indicates enrichment;

2. Number of nts gives the size of the nucleotide block for which the strongest enrichment was observed.

Supplementary Table 4

Summary of feature enrichment analysis for V-PAR-CLIP data

Features	Seed			Seedless		
	3′ UTR	CDS	5′ UTR	3′ UTR	CDS	5′ UTR
ΔG_{total}	•	•	•	•	•	•
ΔG_{hybrid}						
ΔG_{nucl}	•	•	•	•	•	•
Seed type	•	•	•			
miRNA 3′ base-pairing	•	•	•	•	•	•
Site accessibility	•	•	•	•	•	•
Seed accessibility	•	•				
Upstream accessibility	• 10 nt	• 15 nt		• 10 nt	• 15 nt	
Downstream accessibility	• 30 nt	• 10 nt	• 25 nt	• 10 nt	• 15 nt	• 15 nt
Upstream AU content	• 30 nt	• 30 nt	• 25 nt	• 25 nt	• 20 nt	• 25 nt
Downstream AU content	• 30 nt	• 25 nt	• 30 nt	• 30 nt	• 20 nt	• 30 nt
Site conservation score	•	•		•	•	•
Seed conservation score	•	•				
Site location	•		•	•	•	•

1. • indicates enrichment;

2. Number of nts gives the size of the nucleotide block for which the strongest enrichment was observed.

Supplementary Table 5

Summary of feature enrichment analysis for V-PAR-CLIP-MNase data

Features	Seed			Seedless		
	3′ UTR	CDS	5′ UTR	3′ UTR	CDS	5′ UTR
ΔG_{total}	•	•	•	•	•	•
ΔG_{hybrid}	•	•	•			
ΔG_{nucl}	•	•				
Seed type	•	•	•			
miRNA 3′ base-pairing	•	•	•	•	•	•
Site accessibility		•			•	
Seed accessibility		•				
Upstream accessibility		• 15 nt			• 20 nt	
Downstream accessibility		• 15 nt			• 15 nt	
Upstream AU content	• 20 nt	• 20 nt		• 30 nt	• 20 nt	
Downstream AU content	• 20 nt	• 20 nt		• 30 nt	• 25 nt	
Site conservation score	•	•		•	•	•
Seed conservation score	•	•				
Site location	•	•	•	•	•	•

1. • indicates enrichment;
2. Number of nts gives the size of the nucleotide block for which the strongest enrichment was observed.

Supplementary Figure 1

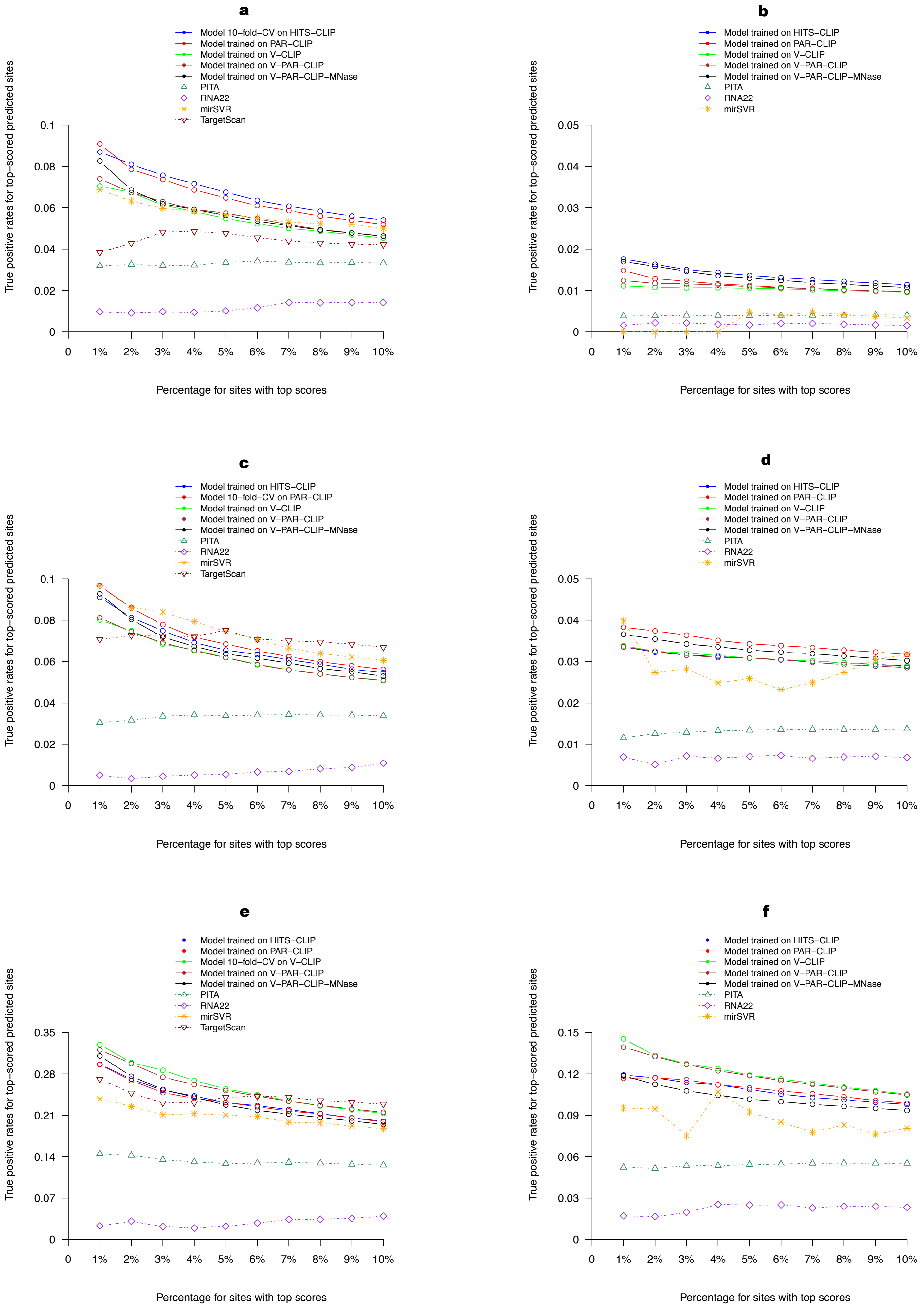

g

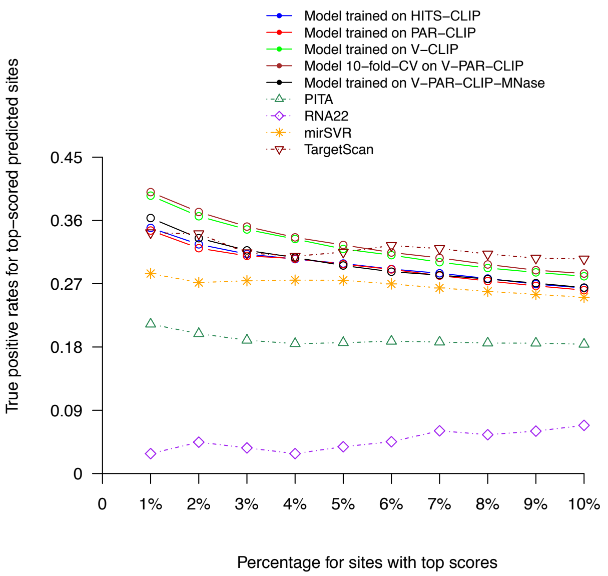

Model trained on HITS−CLIP
Model trained on PAR−CLIP
Model trained on V−CLIP
Model 10−fold−CV on V−PAR−CLIP
Model trained on V−PAR−CLIP−MNase
PITA
RNA22
mirSVR
TargetScan
True positive rates for top−scored predicted sites
0.45
0.36
0.27
0.18
0.09
0
0 1% 2% 3% 4% 5% 6% 7% 8% 9% 10%
Percentage for sites with top scores

h

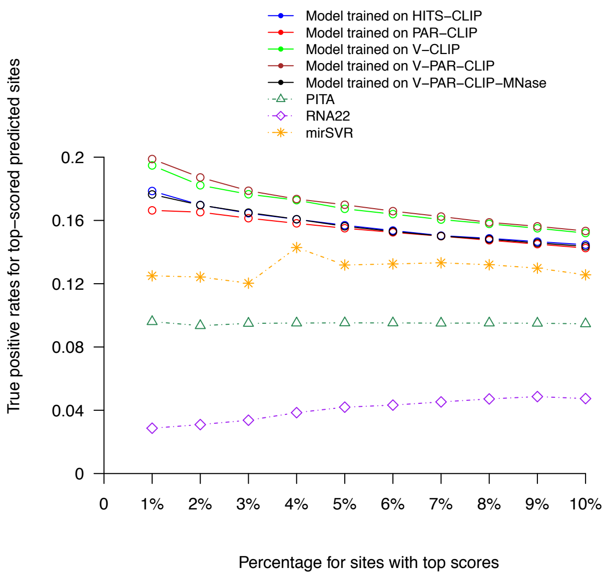

Model trained on HITS−CLIP
Model trained on PAR−CLIP
Model trained on V−CLIP
Model trained on V−PAR−CLIP
Model trained on V−PAR−CLIP−MNase
PITA
RNA22
mirSVR
True positive rates for top−scored predicted sites
0.2
0.16
0.12
0.08
0.04
0
0 1% 2% 3% 4% 5% 6% 7% 8% 9% 10%
Percentage for sites with top scores

i

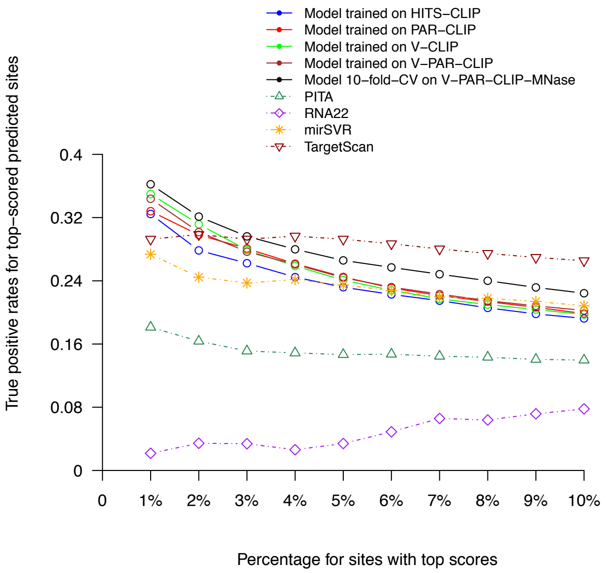

Model trained on HITS−CLIP
Model trained on PAR−CLIP
Model trained on V−CLIP
Model trained on V−PAR−CLIP
Model 10−fold−CV on V−PAR−CLIP−MNase
PITA
RNA22
mirSVR
TargetScan
True positive rates for top−scored predicted sites
0.4
0.32
0.24
0.16
0.08
0
0 1% 2% 3% 4% 5% 6% 7% 8% 9% 10%
Percentage for sites with top scores

j

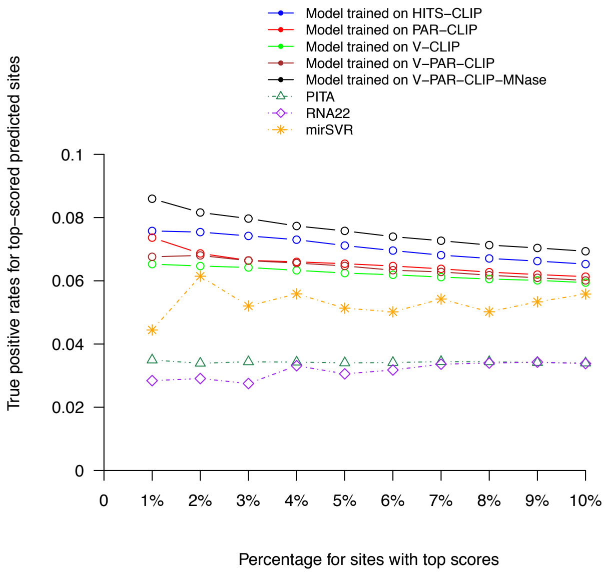

Model trained on HITS−CLIP
Model trained on PAR−CLIP
Model trained on V−CLIP
Model trained on V−PAR−CLIP
Model trained on V−PAR−CLIP−MNase
PITA
RNA22
mirSVR
True positive rates for top−scored predicted sites
0.1
0.08
0.06
0.04
0.02
0
0 1% 2% 3% 4% 5% 6% 7% 8% 9% 10%
Percentage for sites with top scores

Supplementary Figure 2

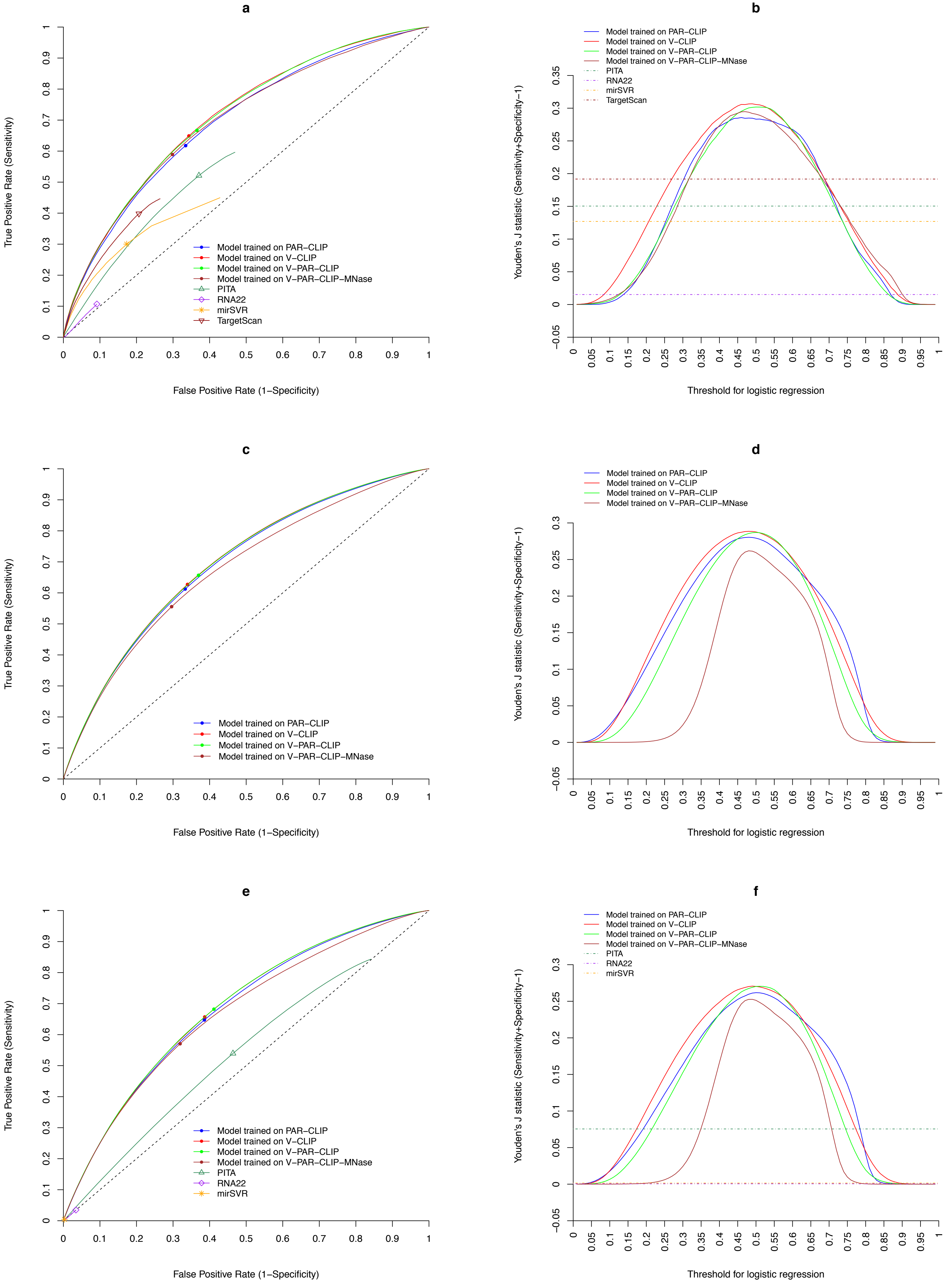

Supplementary Figure 3

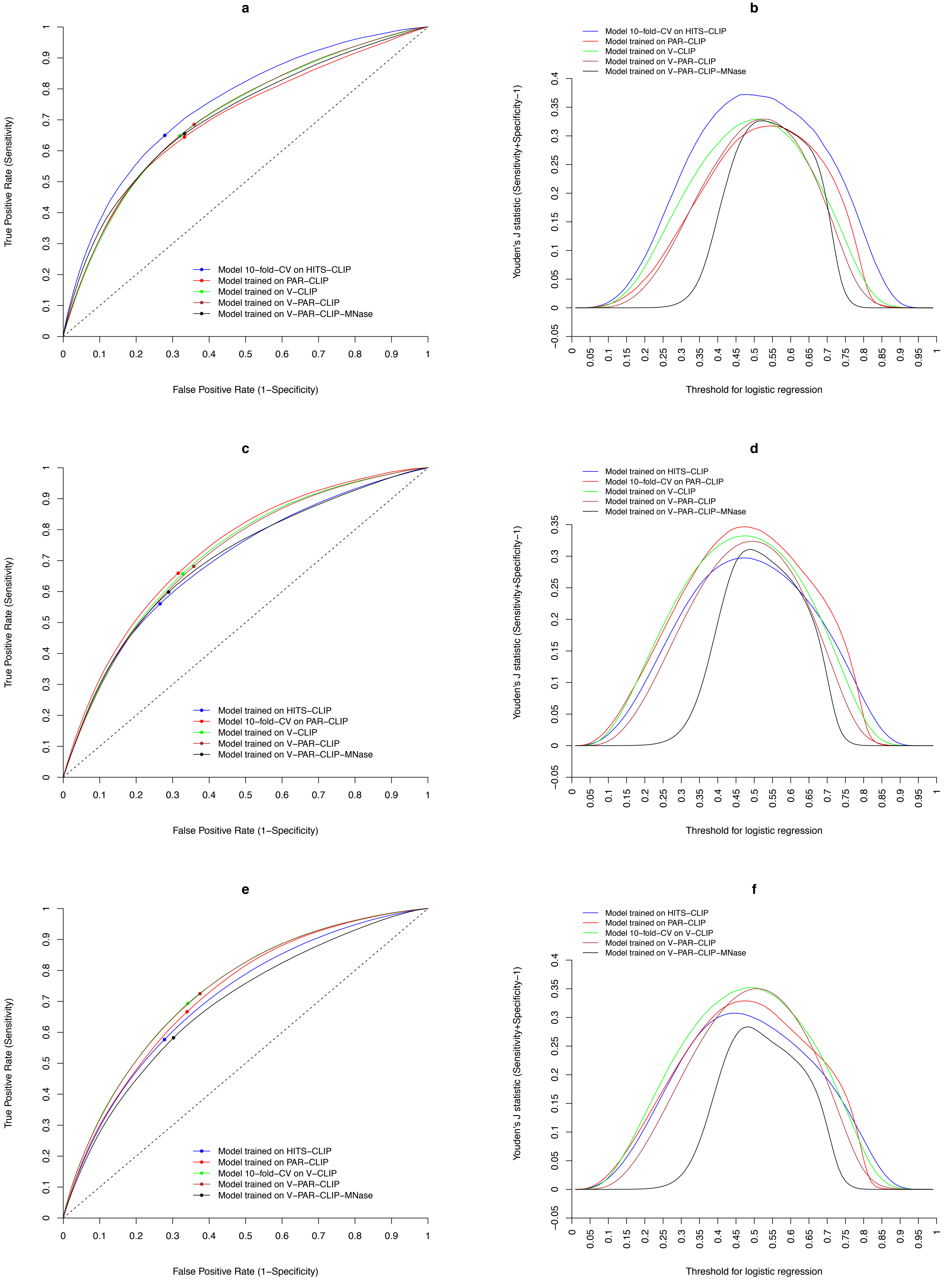

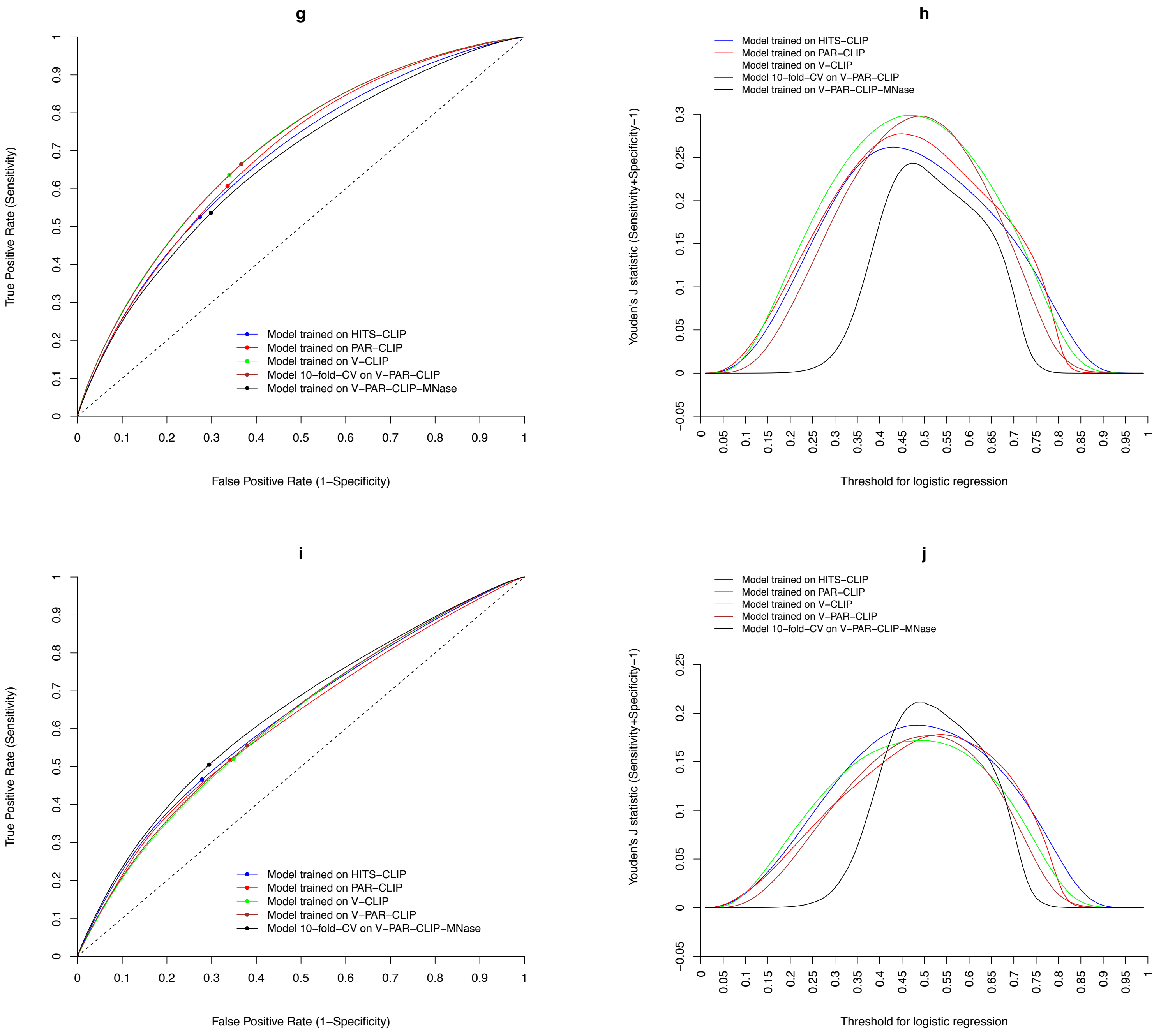
g
True Positive Rate (Sensitivity)
False Positive Rate (1−Specificity)
Model trained on HITS−CLIP
Model trained on PAR−CLIP
Model trained on V−CLIP
Model 10−fold−CV on V−PAR−CLIP
Model trained on V−PAR−CLIP−MNase
h
Model trained on HITS−CLIP
Model trained on PAR−CLIP
Model trained on V−CLIP
Model 10−fold−CV on V−PAR−CLIP
Model trained on V−PAR−CLIP−MNase
Youden's J statistic (Sensitivity+Specificity−1)
Threshold for logistic regression
i
True Positive Rate (Sensitivity)
False Positive Rate (1−Specificity)
Model trained on HITS−CLIP
Model trained on PAR−CLIP
Model trained on V−CLIP
Model trained on V−PAR−CLIP
Model 10−fold−CV on V−PAR−CLIP−MNase
j
Model trained on HITS−CLIP
Model trained on PAR−CLIP
Model trained on V−CLIP
Model trained on V−PAR−CLIP
Model 10−fold−CV on V−PAR−CLIP−MNase
Youden's J statistic (Sensitivity+Specificity−1)
Threshold for logistic regression

Supplementary Figure 4

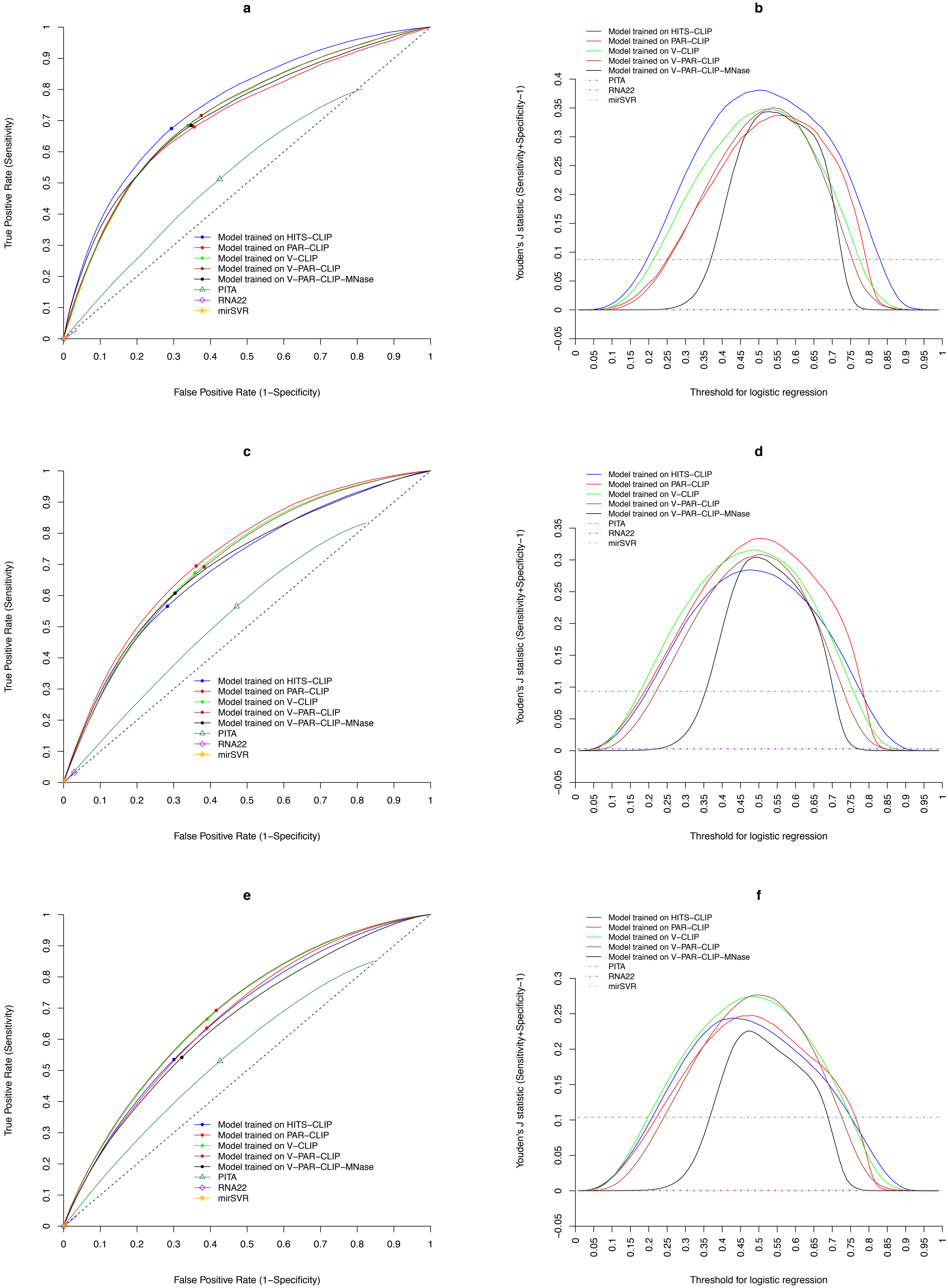

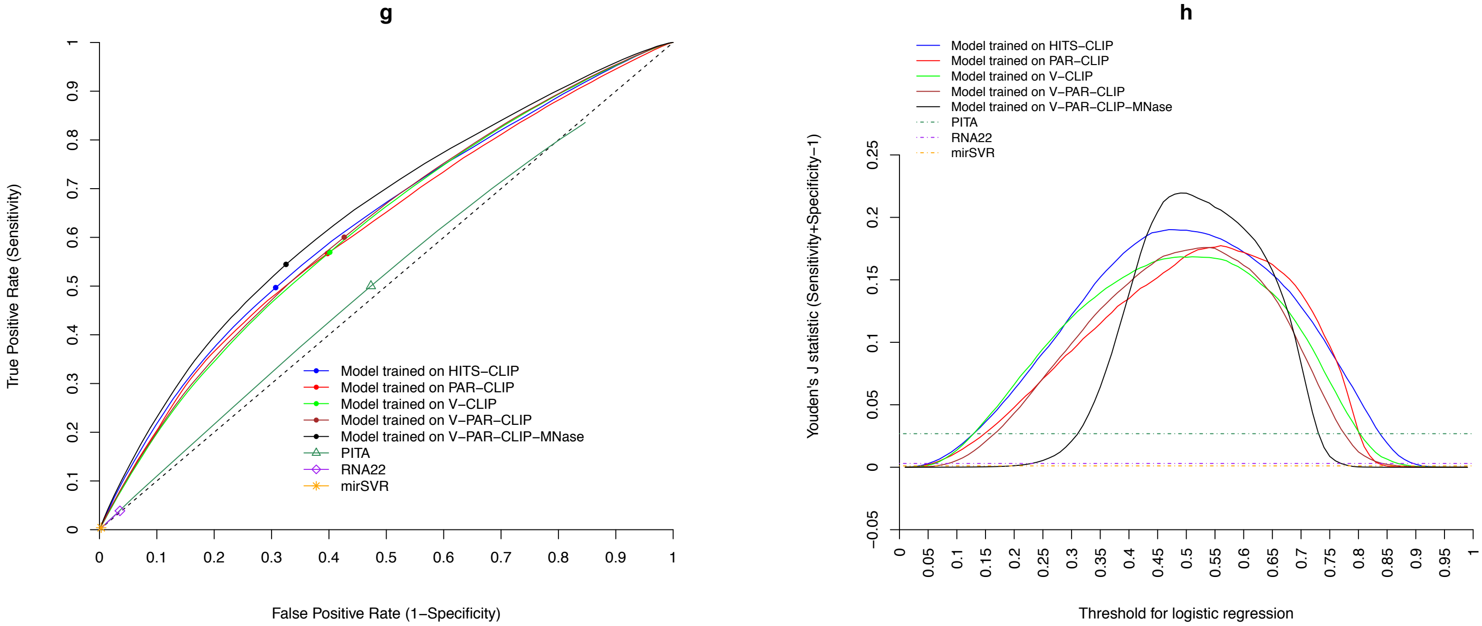
g
True Positive Rate (Sensitivity)
False Positive Rate (1−Specificity)
Model trained on HITS−CLIP
Model trained on PAR−CLIP
Model trained on V−CLIP
Model trained on V−PAR−CLIP
Model trained on V−PAR−CLIP−MNase
PITA
RNA22
mirSVR
h
Youden's J statistic (Sensitivity+Specificity−1)
Threshold for logistic regression
Model trained on HITS−CLIP
Model trained on PAR−CLIP
Model trained on V−CLIP
Model trained on V−PAR−CLIP
Model trained on V−PAR−CLIP−MNase
PITA
RNA22
mirSVR

Supplementary Figure 5

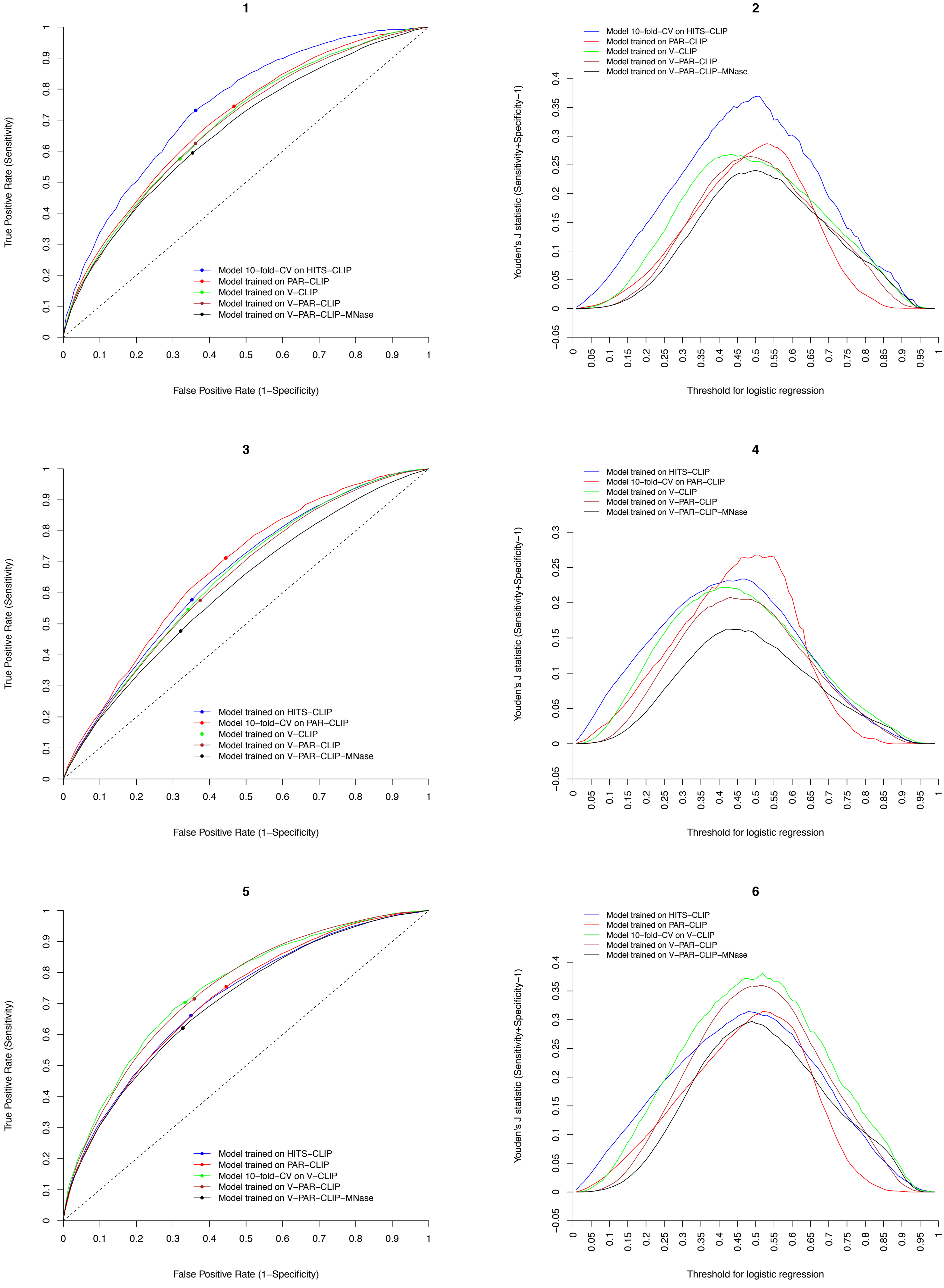

7

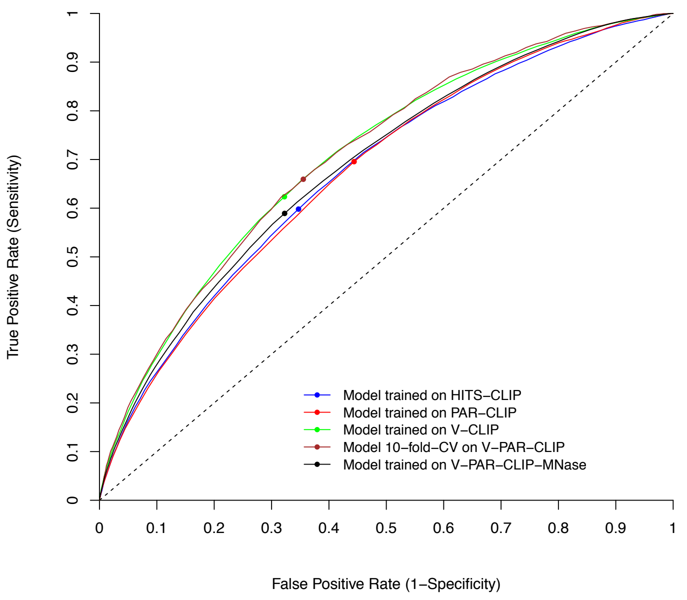
True Positive Rate (Sensitivity)
False Positive Rate (1−Specificity)
Model trained on HITS−CLIP
Model trained on PAR−CLIP
Model trained on V−CLIP
Model 10−fold−CV on V−PAR−CLIP
Model trained on V−PAR−CLIP−MNase

8

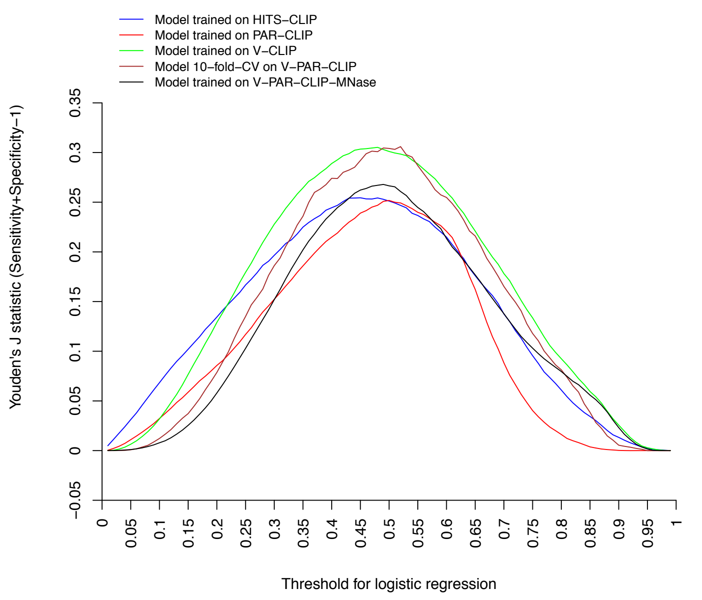
Model trained on HITS−CLIP
Model trained on PAR−CLIP
Model trained on V−CLIP
Model 10−fold−CV on V−PAR−CLIP
Model trained on V−PAR−CLIP−MNase
Youden's J statistic (Sensitivity+Specificity−1)
Threshold for logistic regression

9

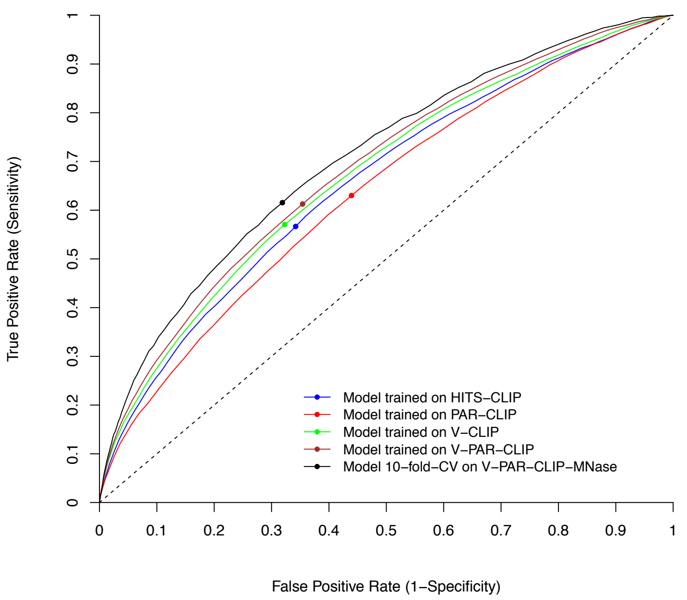
True Positive Rate (Sensitivity)
False Positive Rate (1−Specificity)
Model trained on HITS−CLIP
Model trained on PAR−CLIP
Model trained on V−CLIP
Model trained on V−PAR−CLIP
Model 10−fold−CV on V−PAR−CLIP−MNase

10

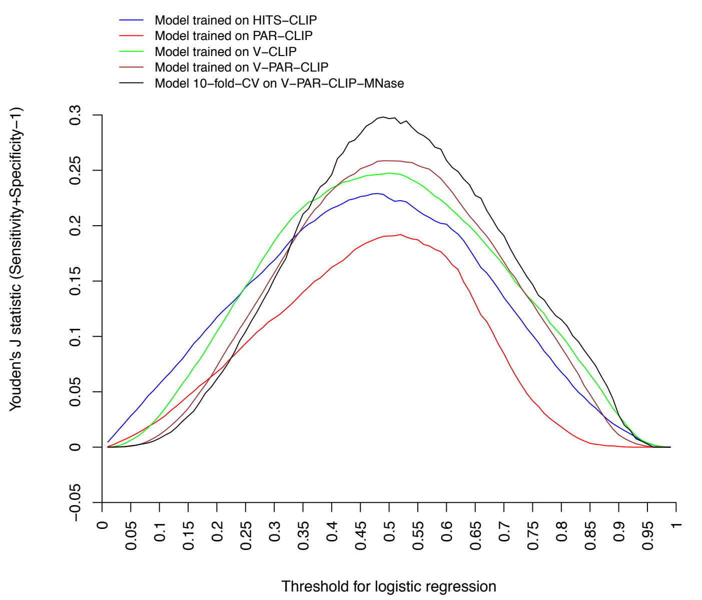
Model trained on HITS−CLIP
Model trained on PAR−CLIP
Model trained on V−CLIP
Model trained on V−PAR−CLIP
Model 10−fold−CV on V−PAR−CLIP−MNase
Youden's J statistic (Sensitivity+Specificity−1)
Threshold for logistic regression

11

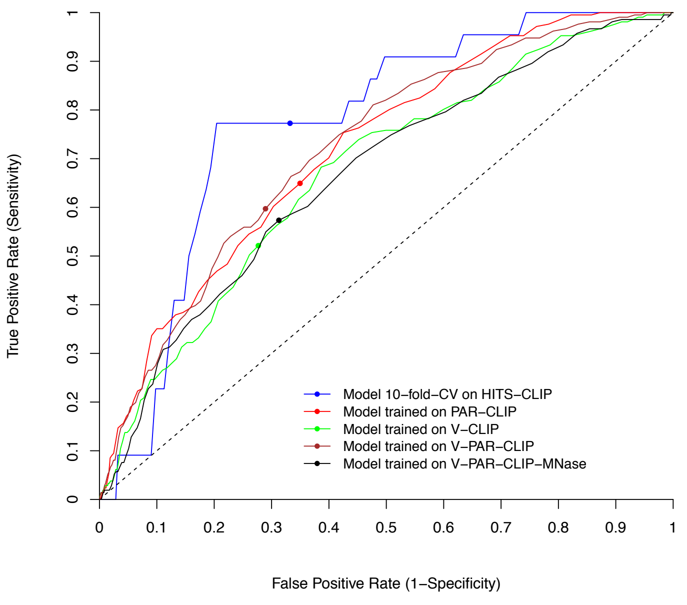
True Positive Rate (Sensitivity)
False Positive Rate (1−Specificity)
Model 10−fold−CV on HITS−CLIP
Model trained on PAR−CLIP
Model trained on V−CLIP
Model trained on V−PAR−CLIP
Model trained on V−PAR−CLIP−MNase

12

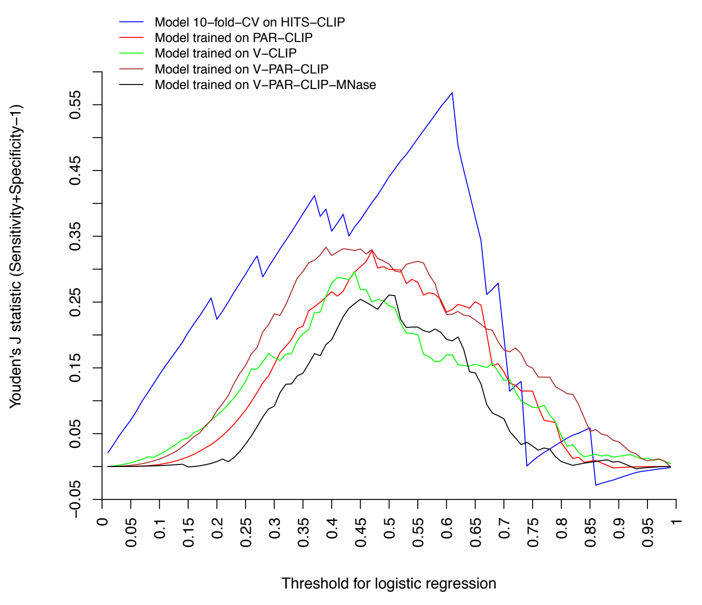
Model 10−fold−CV on HITS−CLIP
Model trained on PAR−CLIP
Model trained on V−CLIP
Model trained on V−PAR−CLIP
Model trained on V−PAR−CLIP−MNase
Youden's J statistic (Sensitivity+Specificity−1)
Threshold for logistic regression

13

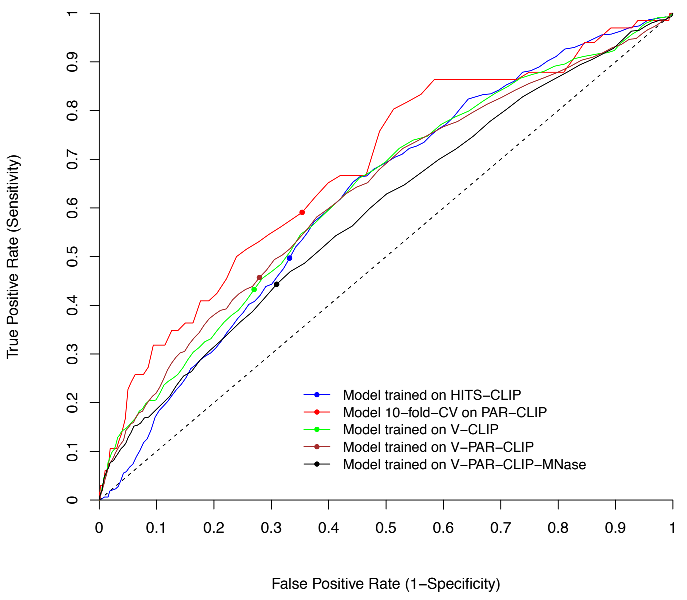

14

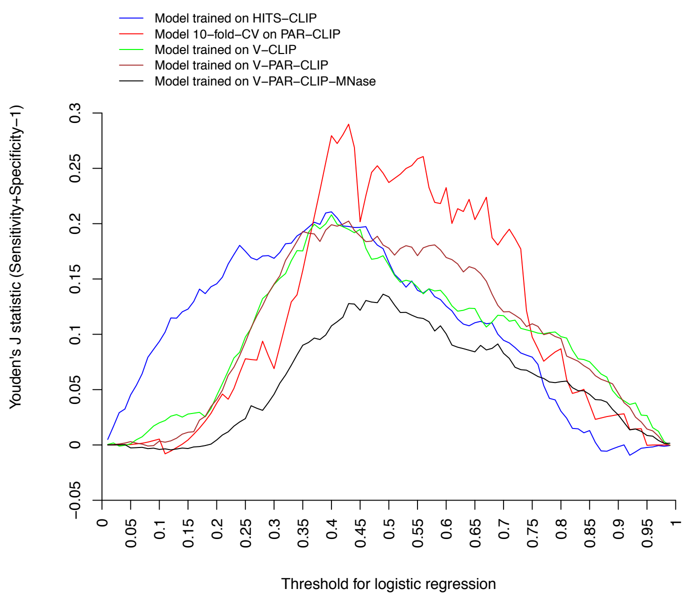

15

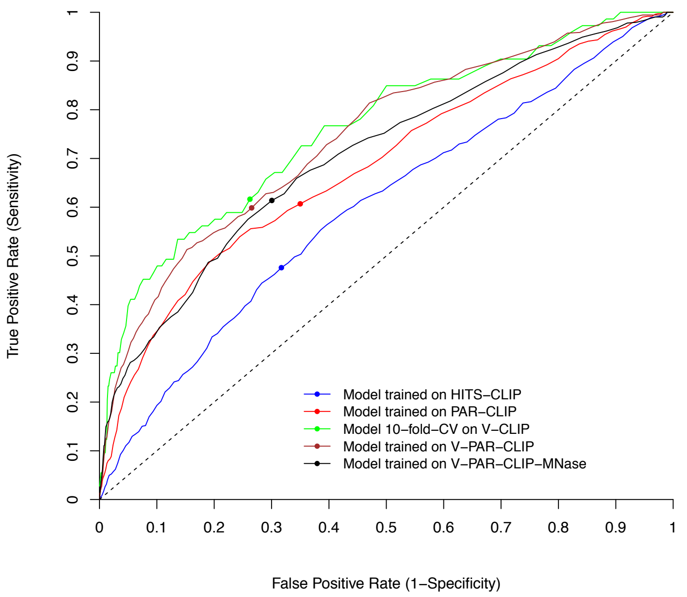

16

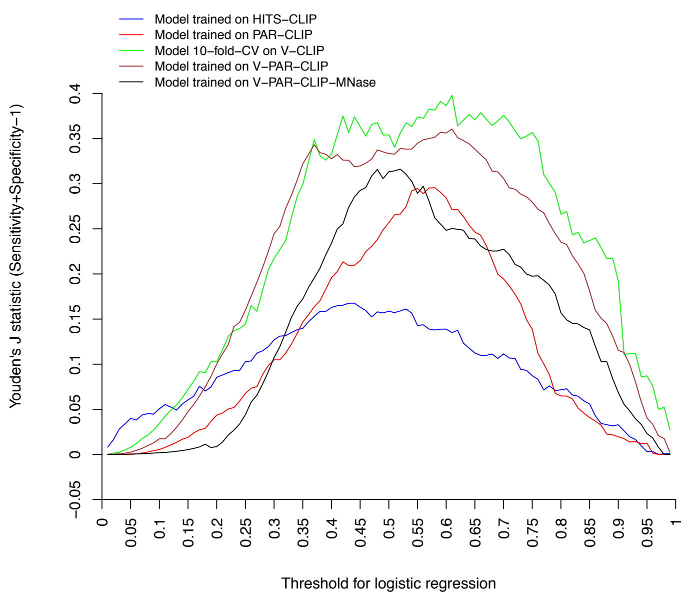

17

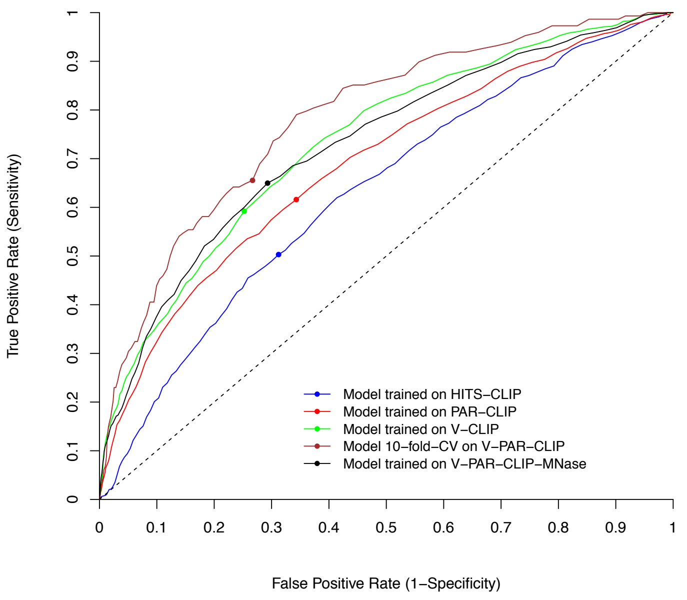

18

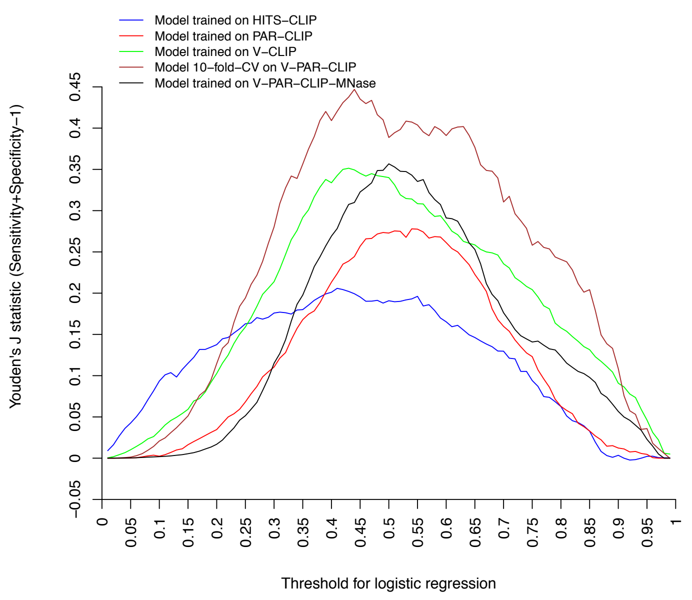

19

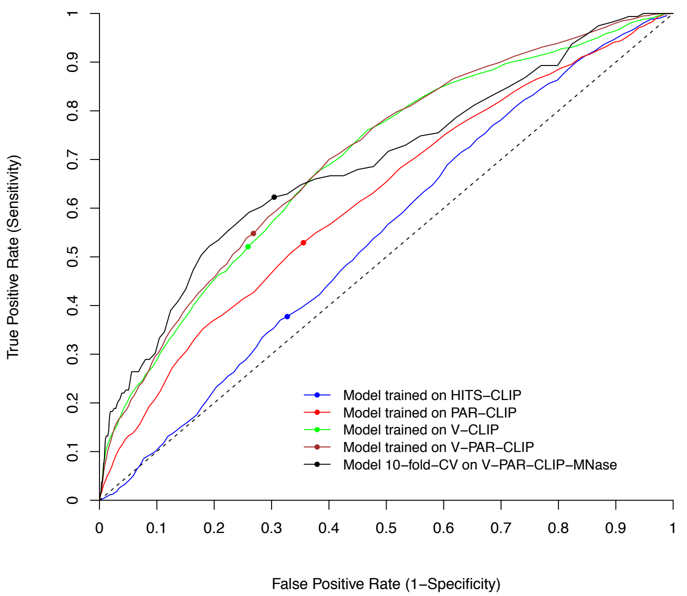

20

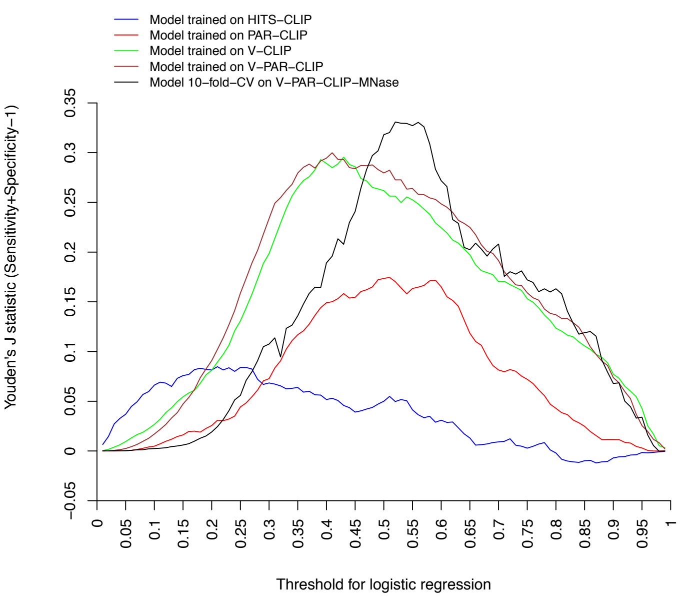

21

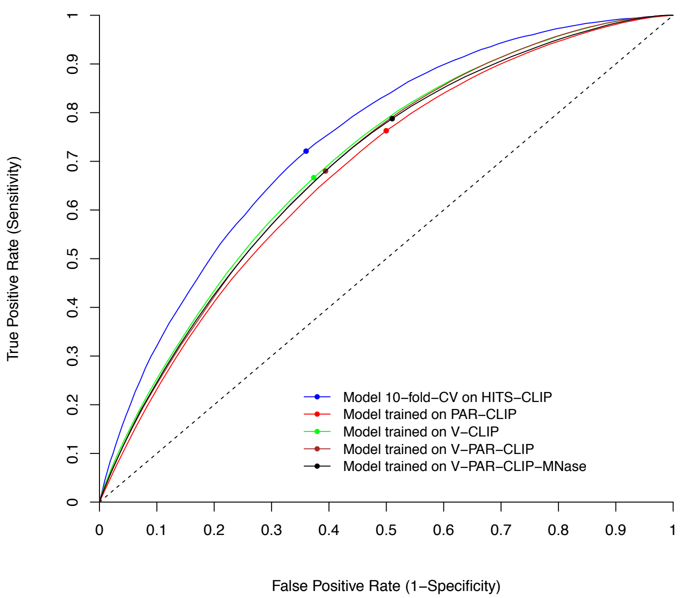

22

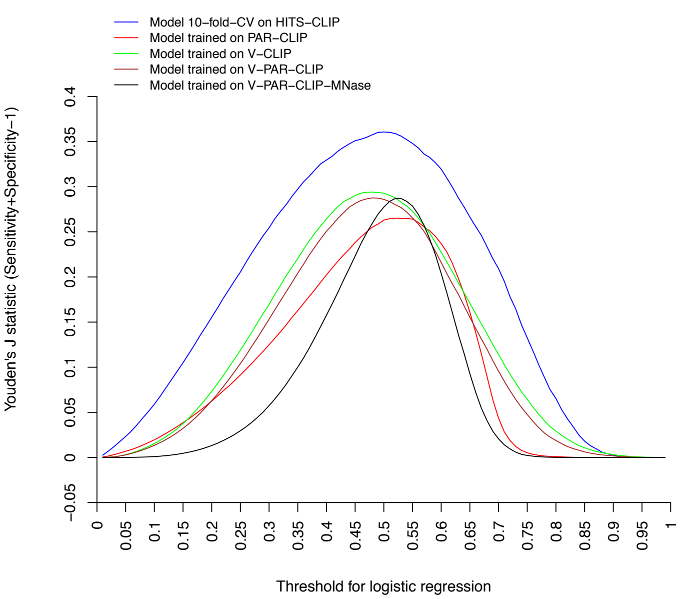

23

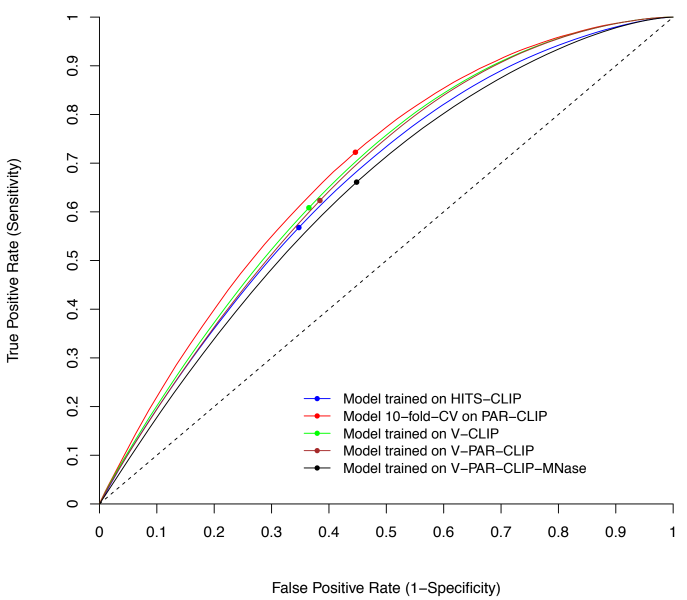

24

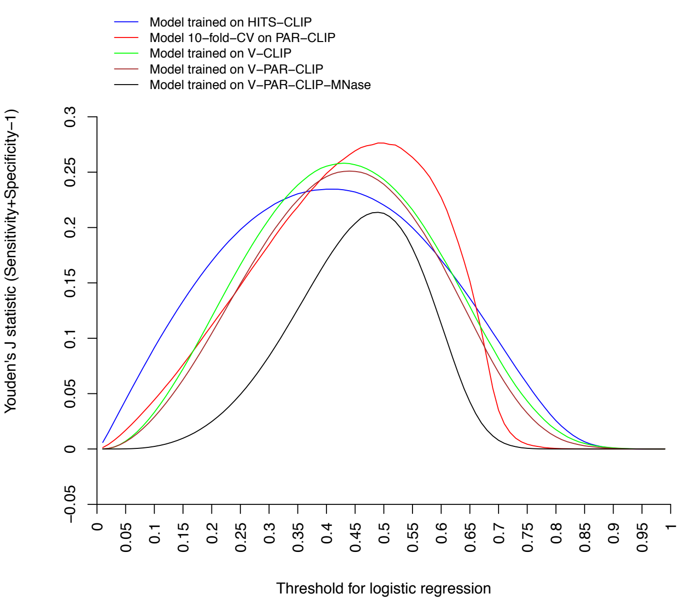

25

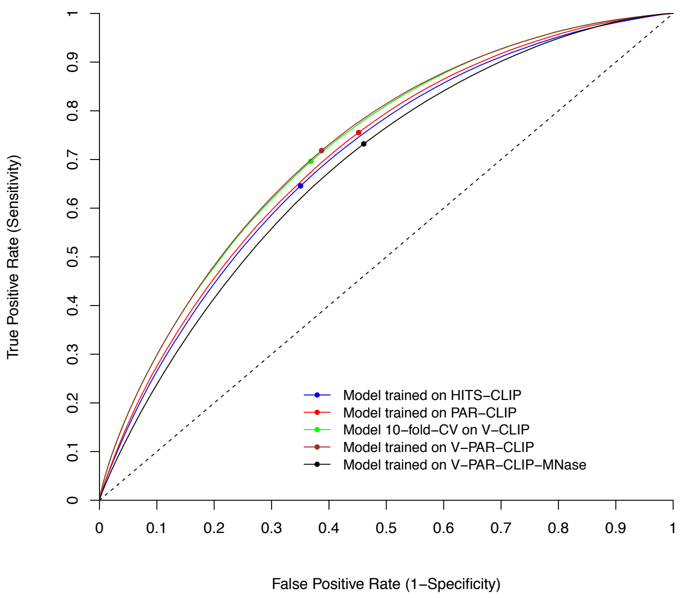

26

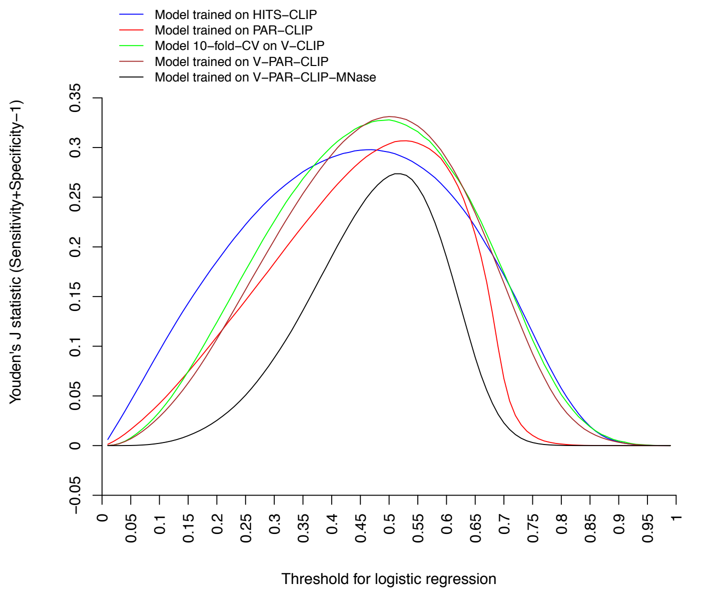

27

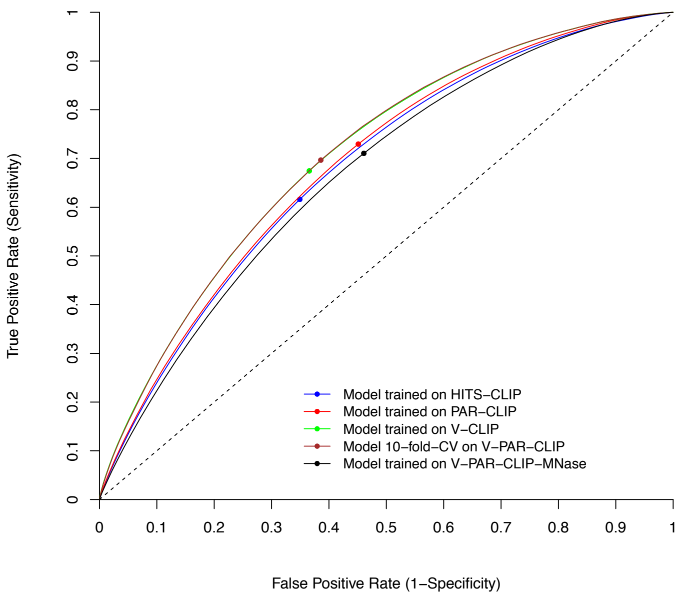

28

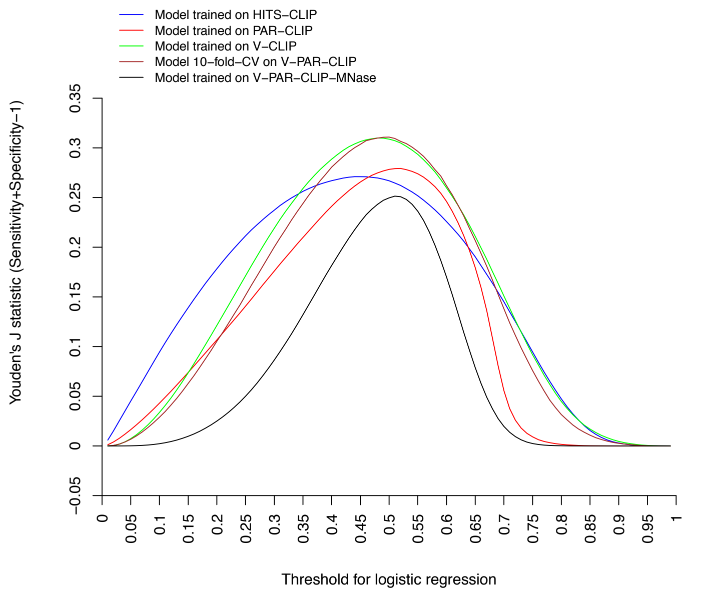

29

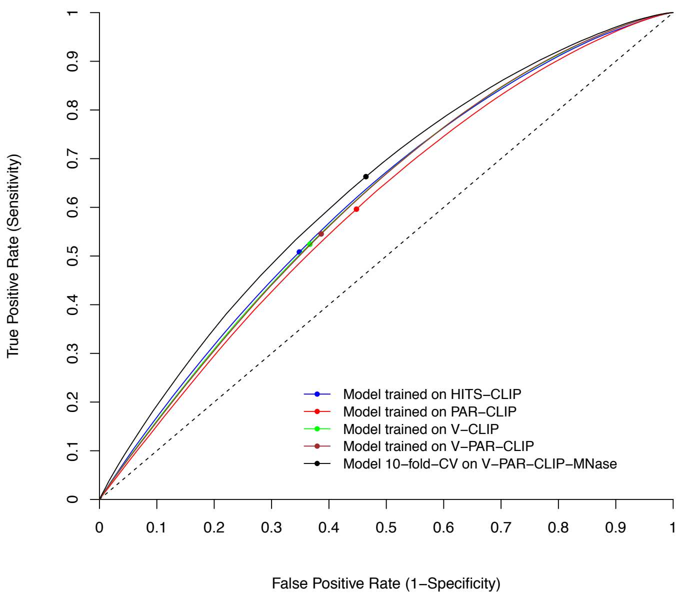

30

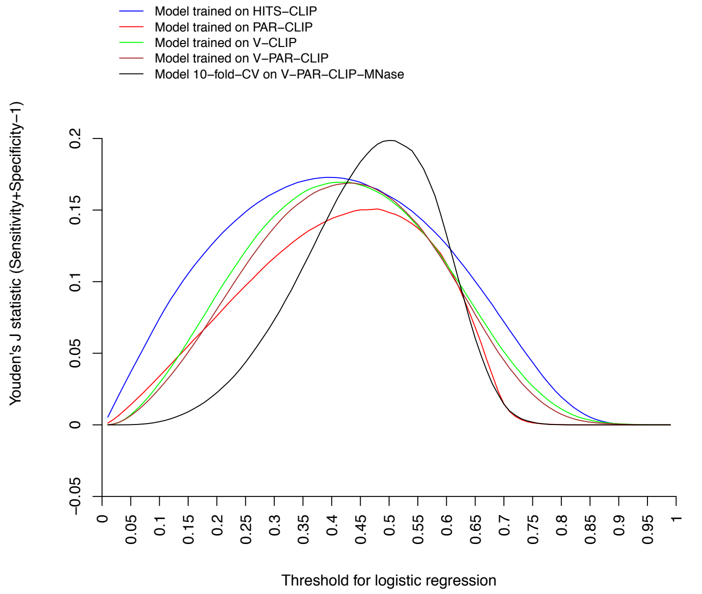

31

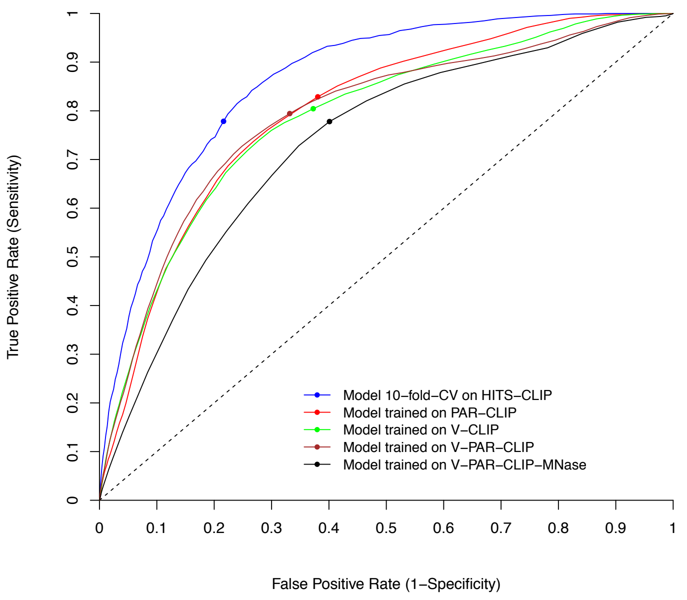

32

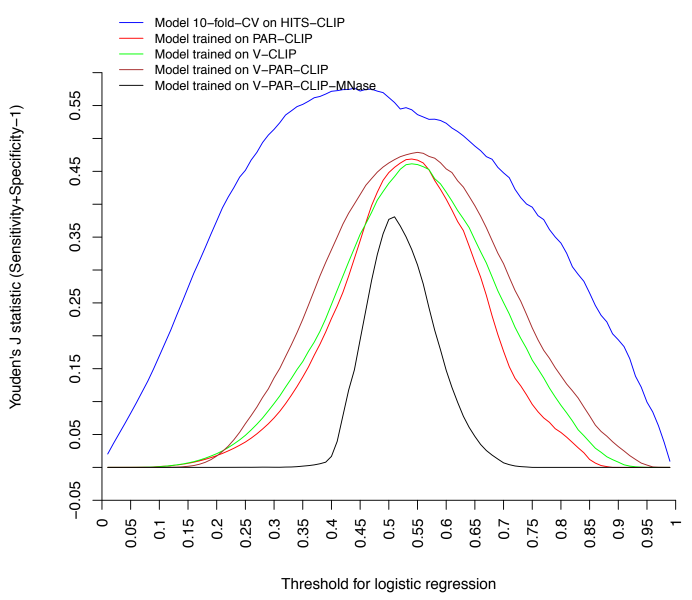

33

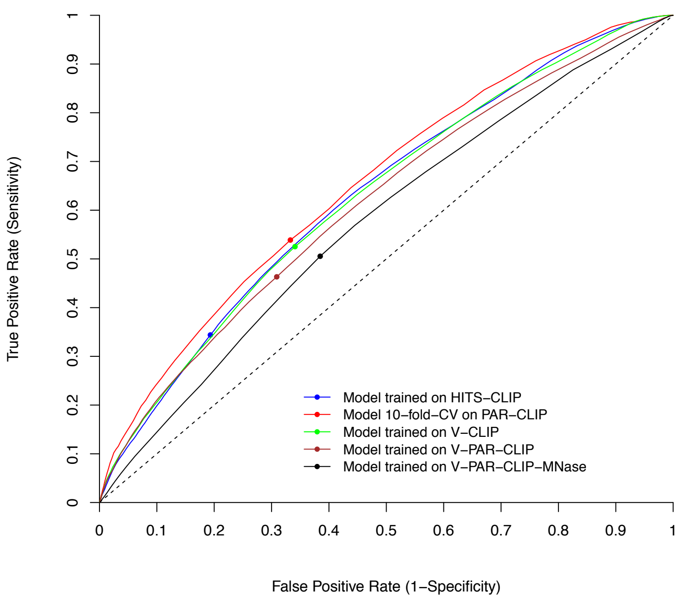

34

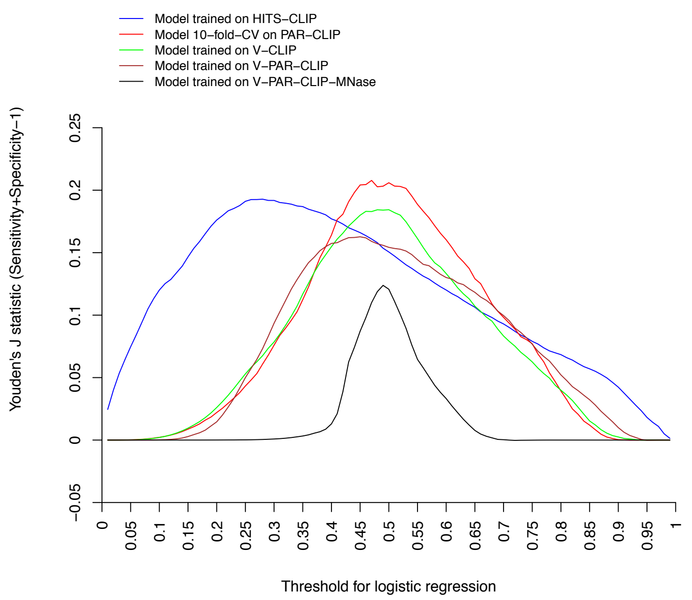

35

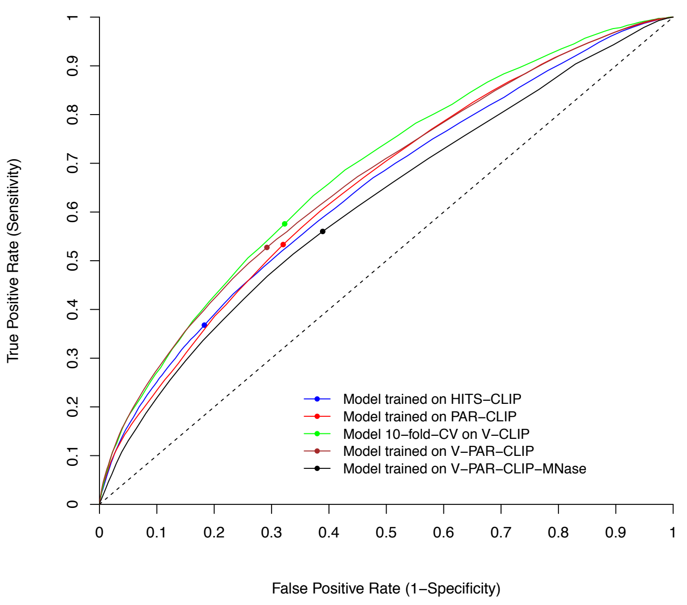

36

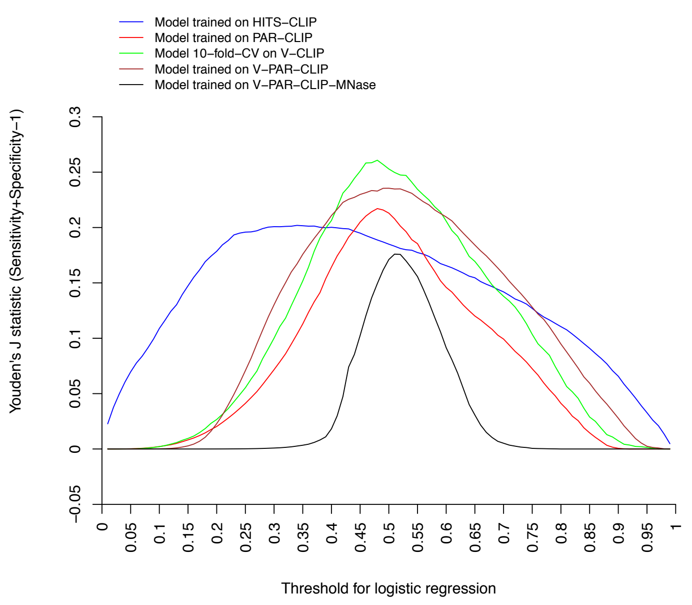

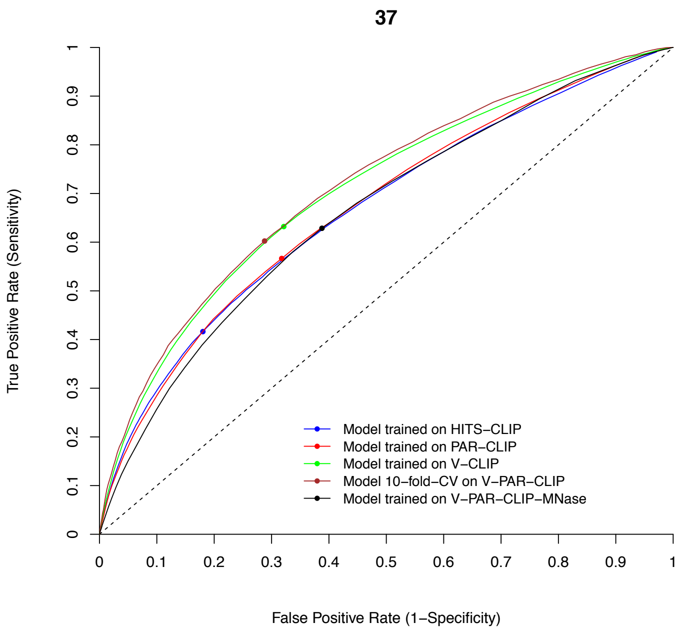
37
True Positive Rate (Sensitivity)
False Positive Rate (1−Specificity)
Model trained on HITS−CLIP
Model trained on PAR−CLIP
Model trained on V−CLIP
Model 10−fold−CV on V−PAR−CLIP
Model trained on V−PAR−CLIP−MNase

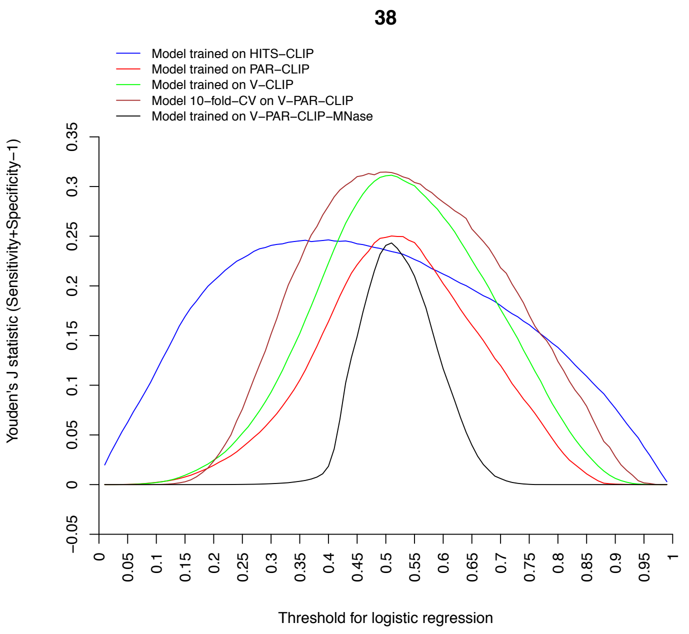
38
Model trained on HITS−CLIP
Model trained on PAR−CLIP
Model trained on V−CLIP
Model 10−fold−CV on V−PAR−CLIP
Model trained on V−PAR−CLIP−MNase
Youden's J statistic (Sensitivity+Specificity−1)
Threshold for logistic regression

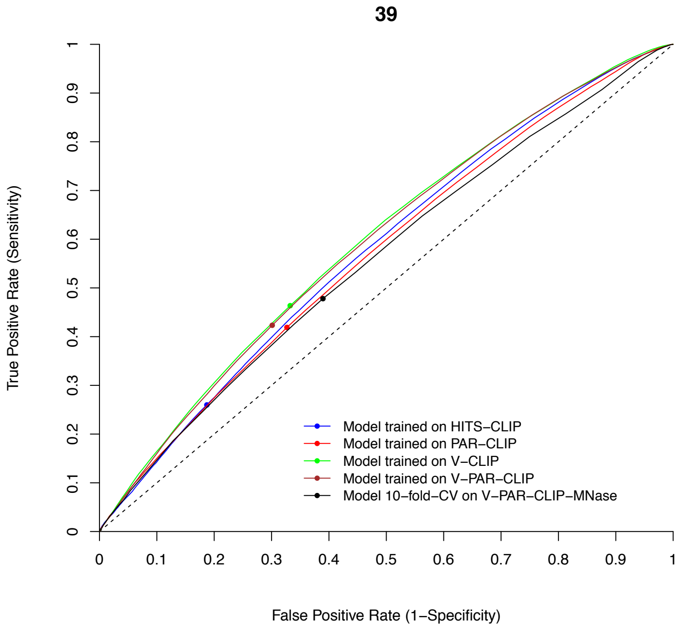
39
True Positive Rate (Sensitivity)
False Positive Rate (1−Specificity)
Model trained on HITS−CLIP
Model trained on PAR−CLIP
Model trained on V−CLIP
Model trained on V−PAR−CLIP
Model 10−fold−CV on V−PAR−CLIP−MNase

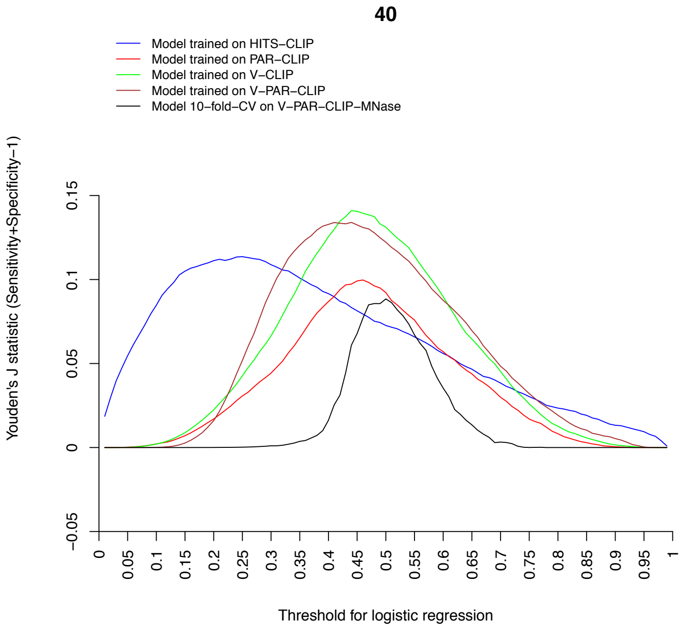
40
Model trained on HITS−CLIP
Model trained on PAR−CLIP
Model trained on V−CLIP
Model trained on V−PAR−CLIP
Model 10−fold−CV on V−PAR−CLIP−MNase
Youden's J statistic (Sensitivity+Specificity−1)
Threshold for logistic regression

Supplementary Figure 6

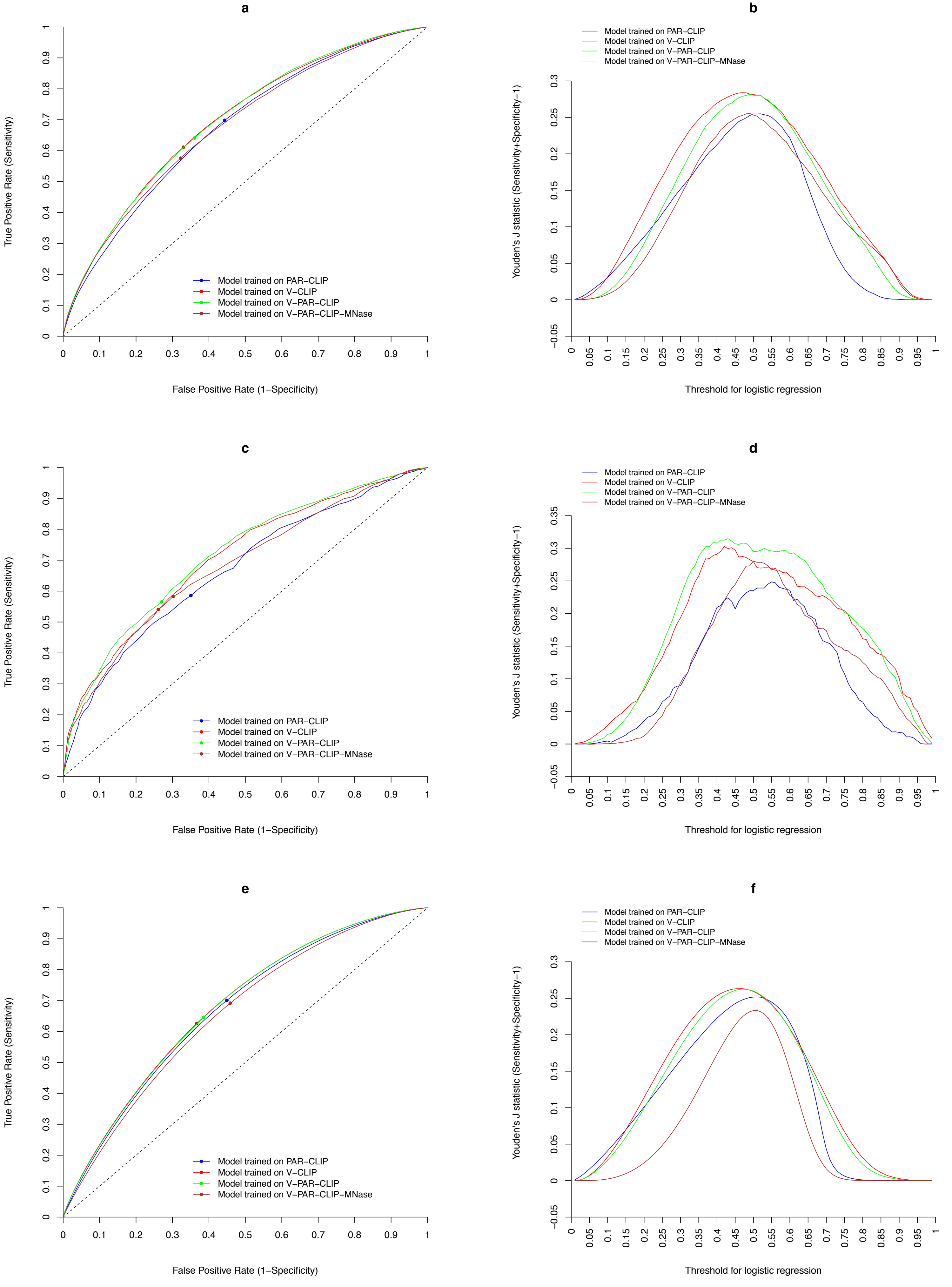

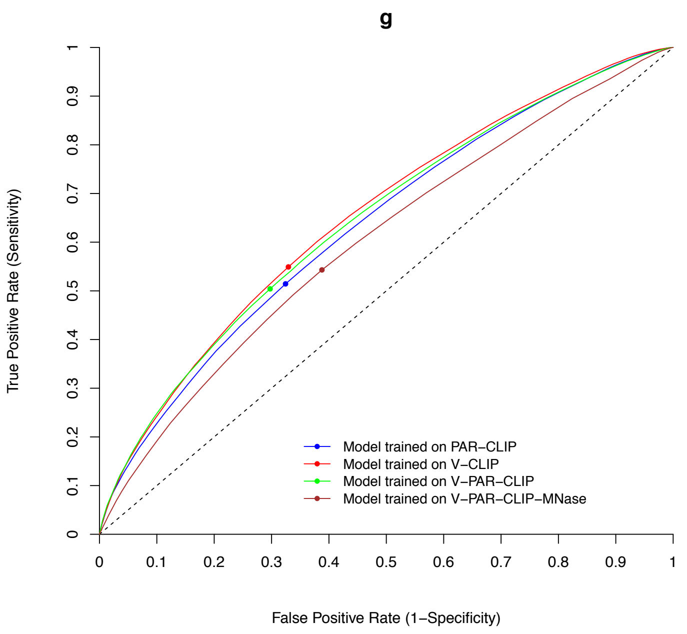
g
True Positive Rate (Sensitivity)
False Positive Rate (1−Specificity)
Model trained on PAR−CLIP
Model trained on V−CLIP
Model trained on V−PAR−CLIP
Model trained on V−PAR−CLIP−MNase
0
0.1
0.2
0.3
0.4
0.5
0.6
0.7
0.8
0.9
1

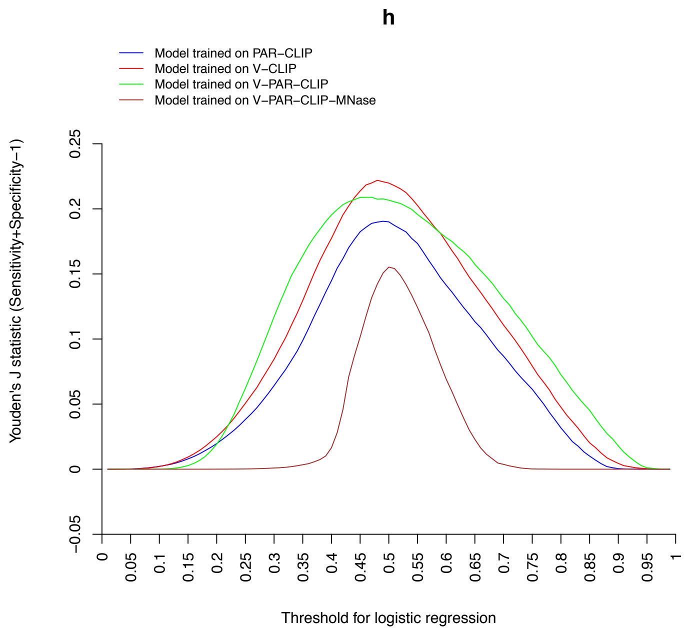
h
Model trained on PAR−CLIP
Model trained on V−CLIP
Model trained on V−PAR−CLIP
Model trained on V−PAR−CLIP−MNase
Youden's J statistic (Sensitivity+Specificity−1)
Threshold for logistic regression
−0.05
0
0.05
0.1
0.15
0.2
0.25
0 0.05 0.1 0.15 0.2 0.25 0.3 0.35 0.4 0.45 0.5 0.55 0.6 0.65 0.7 0.75 0.8 0.85 0.9 0.95 1

Supplementary Figure 7

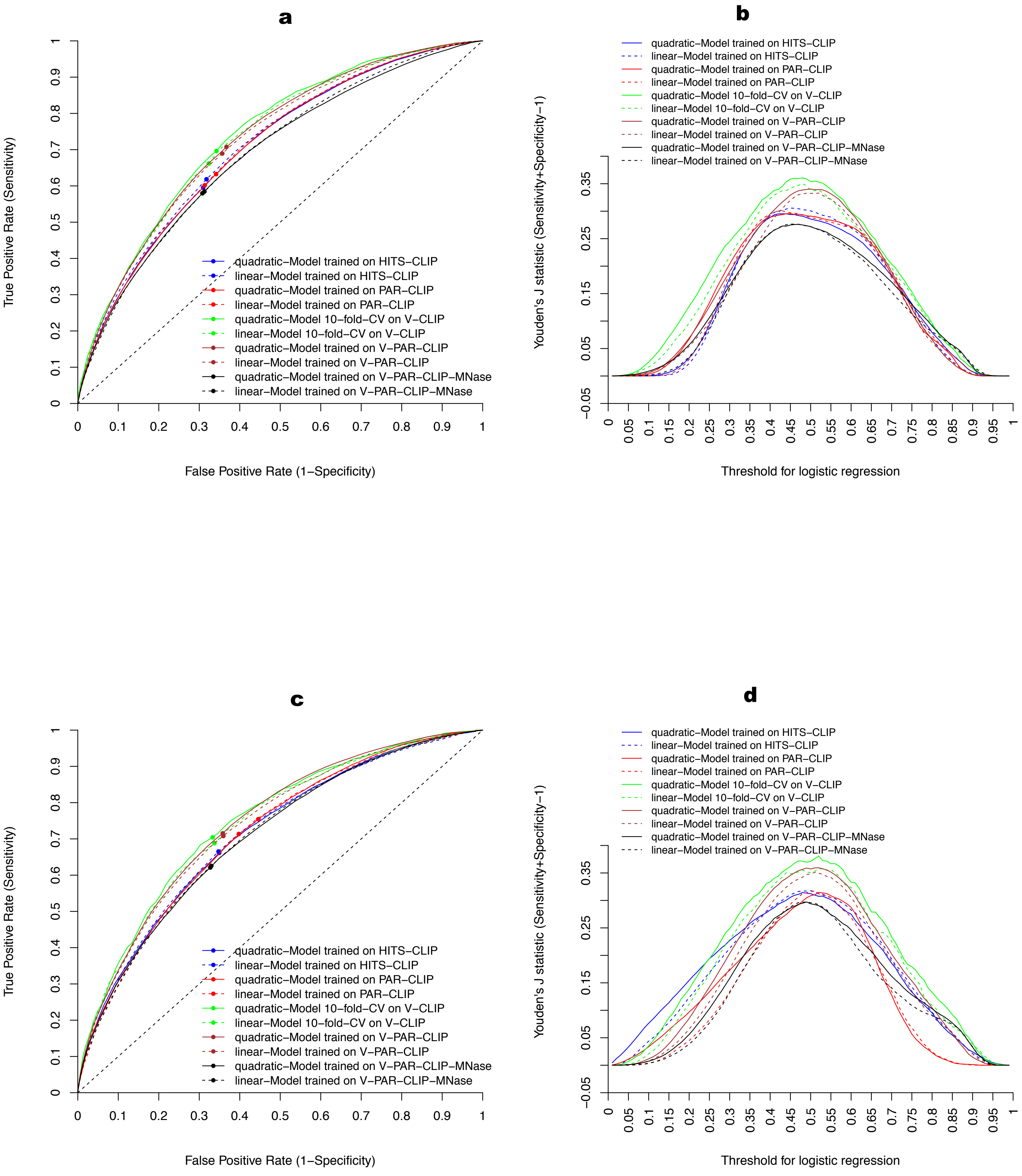

e

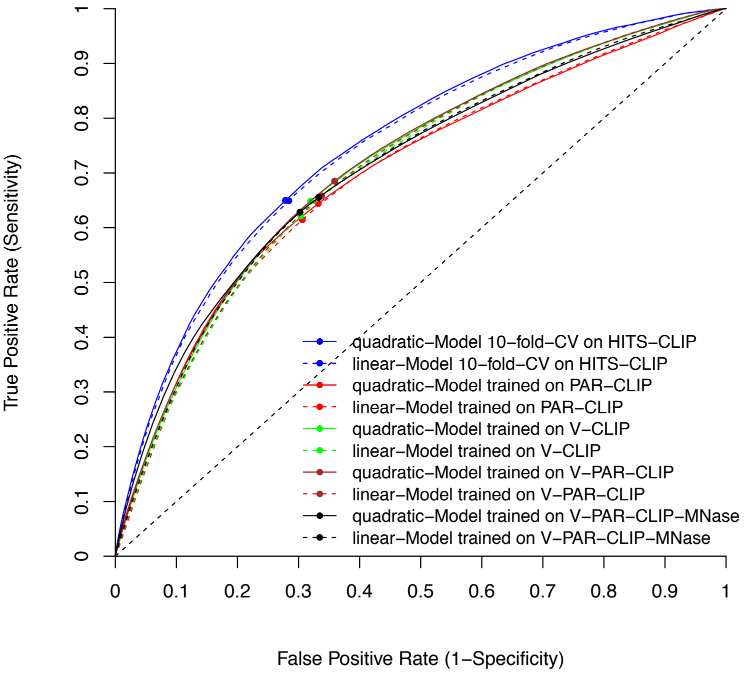

True Positive Rate (Sensitivity)
False Positive Rate (1−Specificity)
quadratic−Model 10−fold−CV on HITS−CLIP
linear−Model 10−fold−CV on HITS−CLIP
quadratic−Model trained on PAR−CLIP
linear−Model trained on PAR−CLIP
quadratic−Model trained on V−CLIP
linear−Model trained on V−CLIP
quadratic−Model trained on V−PAR−CLIP
linear−Model trained on V−PAR−CLIP
quadratic−Model trained on V−PAR−CLIP−MNase
linear−Model trained on V−PAR−CLIP−MNase

f

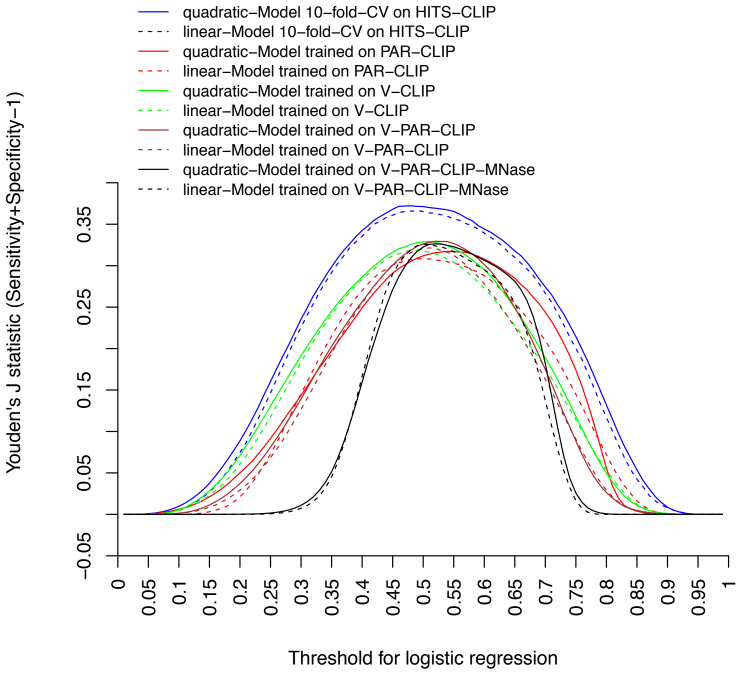

Youden's J statistic (Sensitivity+Specificity−1)
Threshold for logistic regression
quadratic−Model 10−fold−CV on HITS−CLIP
linear−Model 10−fold−CV on HITS−CLIP
quadratic−Model trained on PAR−CLIP
linear−Model trained on PAR−CLIP
quadratic−Model trained on V−CLIP
linear−Model trained on V−CLIP
quadratic−Model trained on V−PAR−CLIP
linear−Model trained on V−PAR−CLIP
quadratic−Model trained on V−PAR−CLIP−MNase
linear−Model trained on V−PAR−CLIP−MNase

g

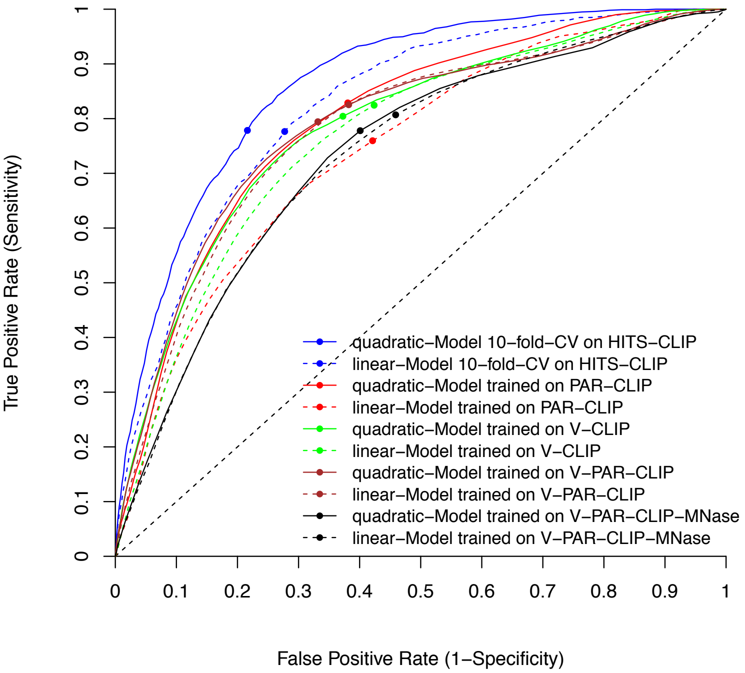

True Positive Rate (Sensitivity)
False Positive Rate (1−Specificity)
quadratic−Model 10−fold−CV on HITS−CLIP
linear−Model 10−fold−CV on HITS−CLIP
quadratic−Model trained on PAR−CLIP
linear−Model trained on PAR−CLIP
quadratic−Model trained on V−CLIP
linear−Model trained on V−CLIP
quadratic−Model trained on V−PAR−CLIP
linear−Model trained on V−PAR−CLIP
quadratic−Model trained on V−PAR−CLIP−MNase
linear−Model trained on V−PAR−CLIP−MNase

h

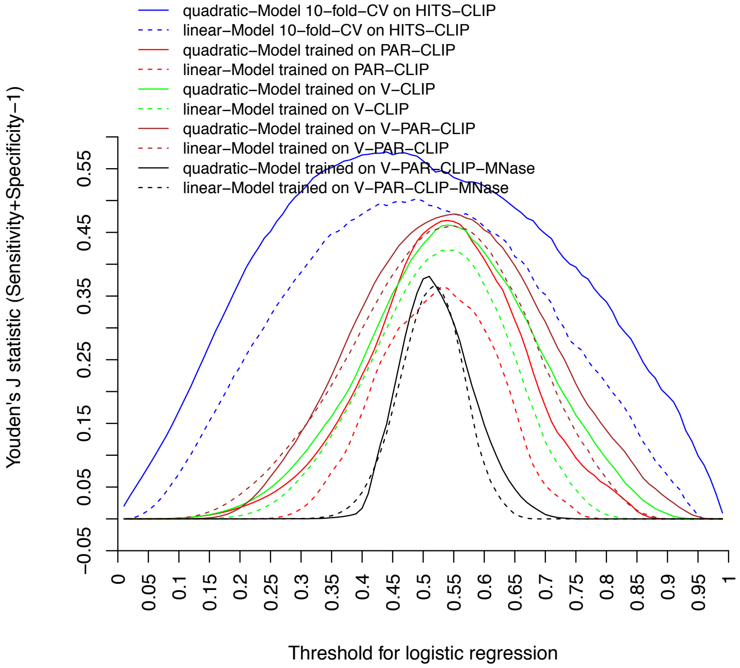

Youden's J statistic (Sensitivity+Specificity−1)
Threshold for logistic regression
quadratic−Model 10−fold−CV on HITS−CLIP
linear−Model 10−fold−CV on HITS−CLIP
quadratic−Model trained on PAR−CLIP
linear−Model trained on PAR−CLIP
quadratic−Model trained on V−CLIP
linear−Model trained on V−CLIP
quadratic−Model trained on V−PAR−CLIP
linear−Model trained on V−PAR−CLIP
quadratic−Model trained on V−PAR−CLIP−MNase
linear−Model trained on V−PAR−CLIP−MNase
